# Stem cell biomanufacturing under uncertainty: A case study in optimizing red blood cell production

**DOI:** 10.1002/aic.16042

**Published:** 2017-12-07

**Authors:** Ruth Misener, Mark C. Allenby, María Fuentes‐Garí, Karan Gupta, Thomas Wiggins, Nicki Panoskaltsis, Efstratios N. Pistikopoulos, Athanasios Mantalaris

**Affiliations:** ^1^ Dept. of Computing Imperial College London South Kensington London SW7 2AZ U.K.; ^2^ Dept. of Haematology Imperial College London Harrow London HA1 3UJ U. K.; ^3^ Artie McFerrin Dept. of Chemical Engineering Texas A&M University College Station TX 77843; ^4^ Dept. of Chemical Engineering Imperial College London South Kensington London SW7 2AZ U.K.

**Keywords:** red blood cell production, bioreactor design under uncertainty, bioprocess optimization under uncertainty, robust optimization, stem cell biomanufacturing

## Abstract

As breakthrough cellular therapy discoveries are translated into reliable, commercializable applications, effective stem cell biomanufacturing requires systematically developing and optimizing bioprocess design and operation. This article proposes a rigorous computational framework for stem cell biomanufacturing under uncertainty. Our mathematical tool kit incorporates: high‐fidelity modeling, single variate and multivariate sensitivity analysis, global topological superstructure optimization, and robust optimization. The advantages of the proposed bioprocess optimization framework using, as a case study, a dual hollow fiber bioreactor producing red blood cells from progenitor cells were quantitatively demonstrated. The optimization phase reduces the cost by a factor of 4, and the price of insuring process performance against uncertainty is approximately 15% over the nominal optimal solution. Mathematical modeling and optimization can guide decision making; the possible commercial impact of this cellular therapy using the disruptive technology paradigm was quantitatively evaluated. © 2017 American Institute of Chemical Engineers *AIChE J*, 64: 3011–3022, 2018

## Introduction

Biomanufacturing process engineering is now at a stage where we can consider systematic production of cell therapies.^1^, ^2^, ^3^, ^4^, ^5^, ^6^, ^7^ But stem cell biomanufacturing is a new industry with high upfront costs and long time scales to market, so commercializing therapies such as *ex vivo* red blood cell (RBC) production requires detailed cost‐benefit analyses and financial planning.^8^ Relevant cost contributions include materials, time, regulatory, and staff. Researchers have begun considering bioprocessing strategies enabling systematic, reliable production of cellular therapies.^1^, ^9^, ^10^, ^11^, ^12^ Mathematical modeling and optimization can positively impact stem cell biomanufacturing.^13^

This article proposes a computational framework for stem cell bioreactor design and operation which: accurately predicts erythropoietic, that is, RBC, maturation in the bioreactor via high‐fidelity modeling, discovers the factors most affecting production/cost/quality through single variate and multivariate sensitivity analysis, determines the best bioreactor design using global topological superstructure optimization, devises operational strategies maximizing bioreactor production under uncertainty via robust optimization, analyzes the likelihood of the bioreactor to be a disruptive technology using net present value (NPV) market analysis. We quantitatively demonstrate the advantages of the proposed bioprocess optimization framework using, as a case study, a dual hollow fiber bioreactor that produces RBC from hematopoietic cell, that is, blood cell, progenitors.^14^, ^15^

This article's core goal is formulating a computational framework facilitating stem cell bioreactor design and operation. The mathematical tool kit builds on algorithms previously developed for mainstream industries, for example, superstructure optimization is frequently applied to petrochemical refining. We show how to adapt, change, and apply the tools toward stem cell bioreactor design. Bioprocess optimization under uncertainty has been previously considered for monoclonal antibody production,^16^, ^17^, ^18^ but mathematical optimization for the cellular therapy industry requires: (1) accurately incorporating cellular kinetics, (2) capturing spatial design scales ranging from individual cellular behavior to bioreactor layout, (3) representing temporal scales ranging from metabolic reactions to cellular differentiation, (4) elucidating the impact uncertainty and heterogeneity have onto the final outcome. This article shows how the proposed computational framework incorporates the four preceding considerations; suggests specific bioreactor features for further experimental study with respect to the target cell type (RBC); motivates the application of this framework to other cellular therapy applications. We also analyze the potential for this to become a disruptive technology, that is, transform an industry with less than 2 years lead time to the clinic.^19^, ^20^

**Figure 1:**
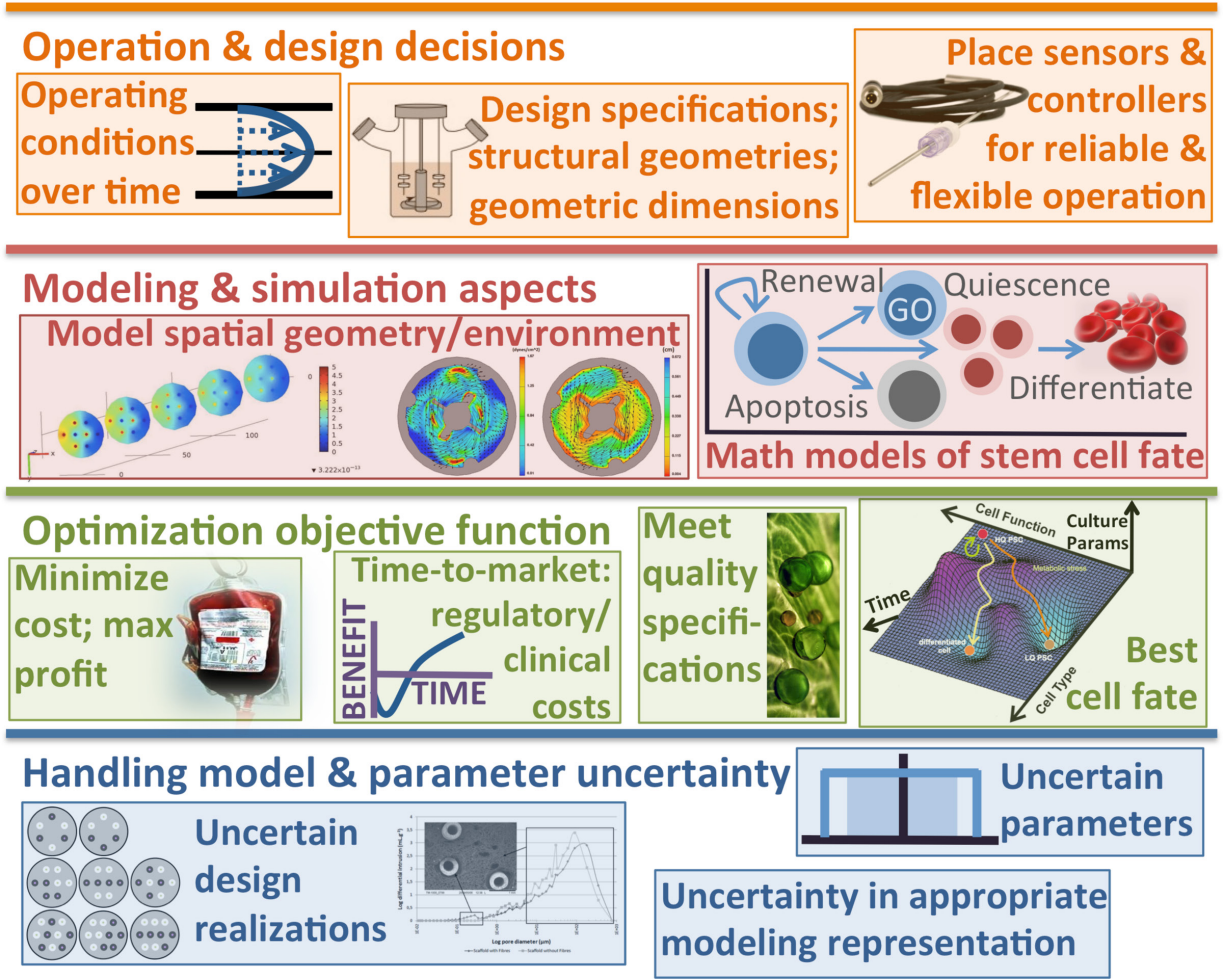
The proposed bioprocess optimization framework consists of: operation and design decisions, modeling and simulation aspects, optimization, and handling model and parameter uncertainty. The recipe illustrated in Figure 2 for building the biomanufacturing framework under uncertainty uses the components in this figure. [Color figure can be viewed at http://wileyonlinelibrary.com]

**Figure 2:**
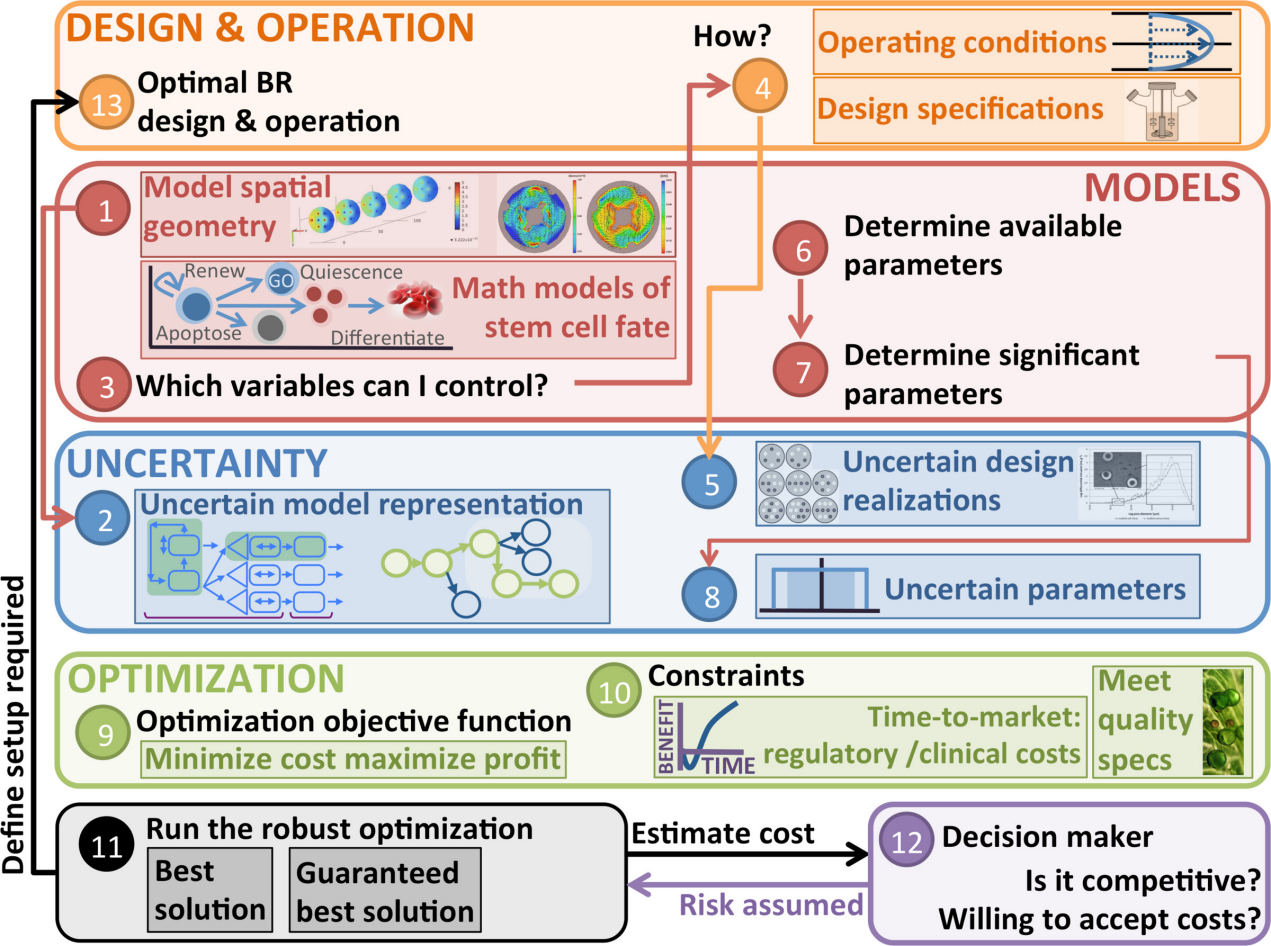
Recipe for the biomanufacturing under uncertainty framework uses the algorithm components illustrated in Figure 1. [Color figure can be viewed at http://wileyonlinelibrary.com]

**Figure 3:**
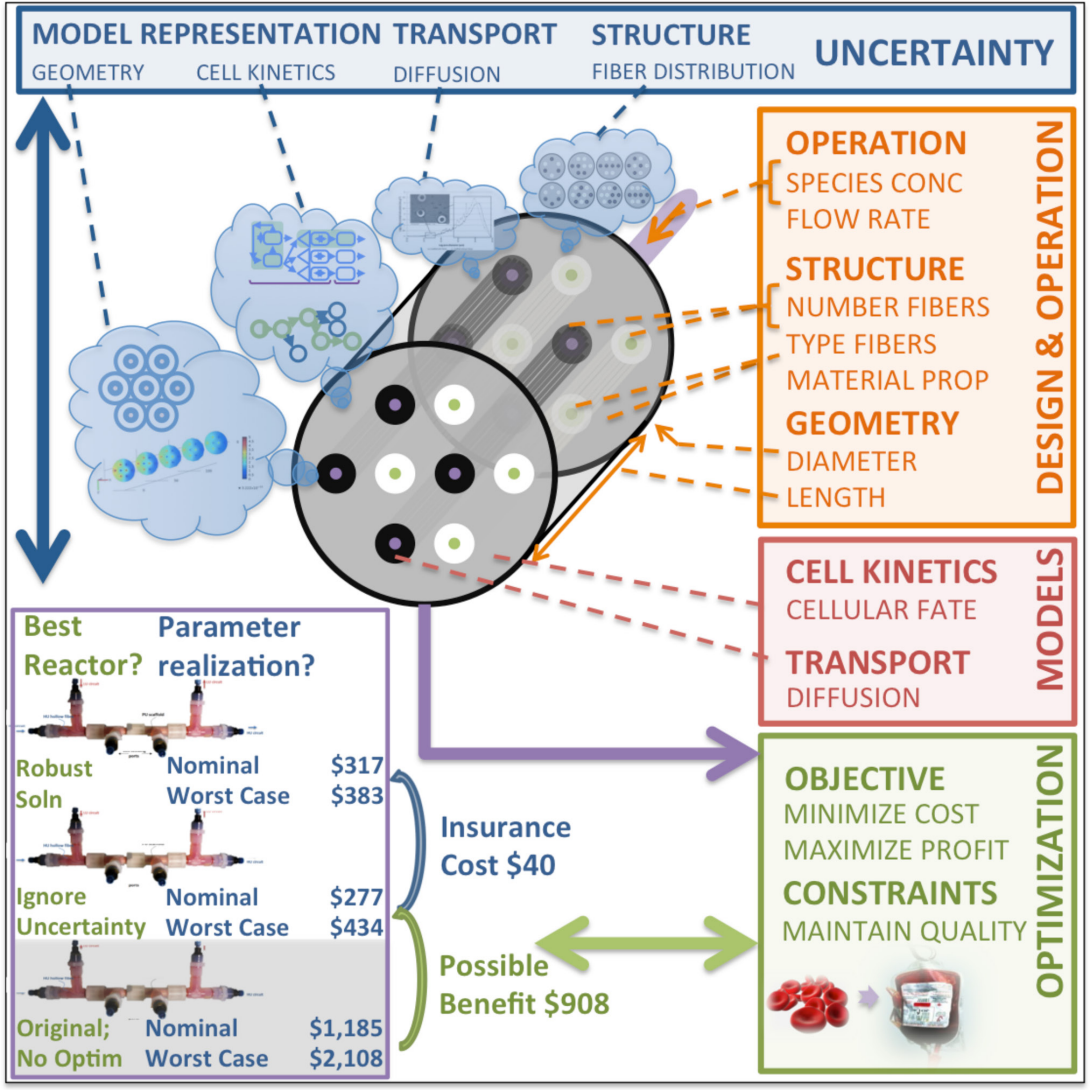
Optimal design under uncertainty for stem cell biomanufacturing requires incorporating four sets of mathematical models: (1) optimization, (2) models of mass transfer and cell kinetics, (3) design and operation, and (4) uncertainty characterization. This application of the methods described in Figure 1 and the recipe diagramed in Figure 2 finds the optimal bioreactor design and operation that works under uncertainty and maximizes the RBCs produced while keeping the cost as low as possible. [Color figure can be viewed at http://wileyonlinelibrary.com]

The modular, mathematical framework being proposed is illustrated in Figures 1, 2, 3. This article belongs to an ongoing effort developing building blocks for modeling and optimizing biomedical systems.^21^, ^22^ Figure 1 diagrams the computational tool kit of six mathematical methods and Figure 2 show their synergies which facilitate stem cell bioreactor design. Figure 3 outlines four sets of mathematical models needed for bioreactor design (objectives, equipment, species, cellular characteristics); each contains its own modeling, optimization, and uncertainty considerations.

**Figure 4:**
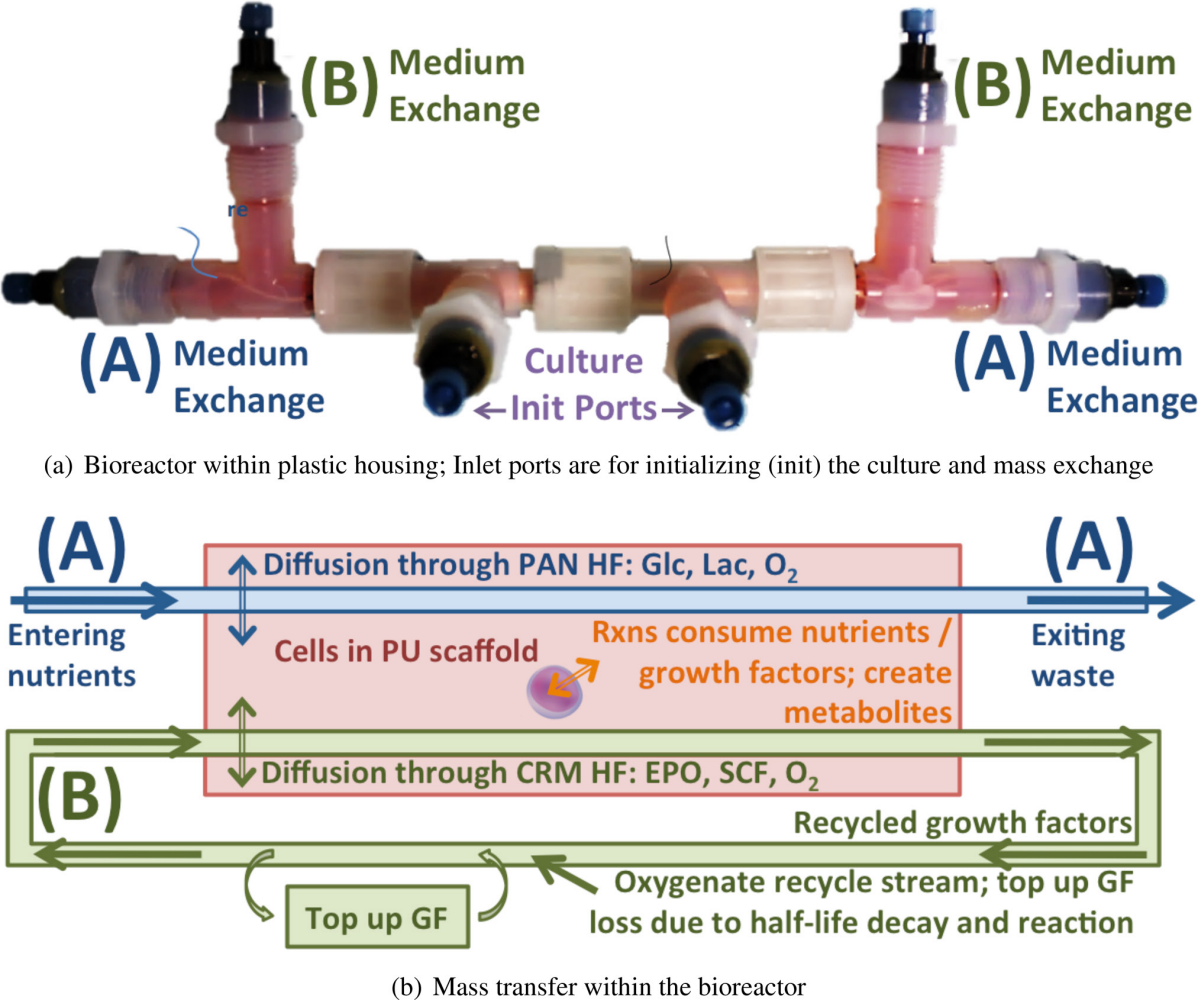
**Bioreactor superstructure.** 15 [Color figure can be viewed at http://wileyonlinelibrary.com]

**Figure 5:**
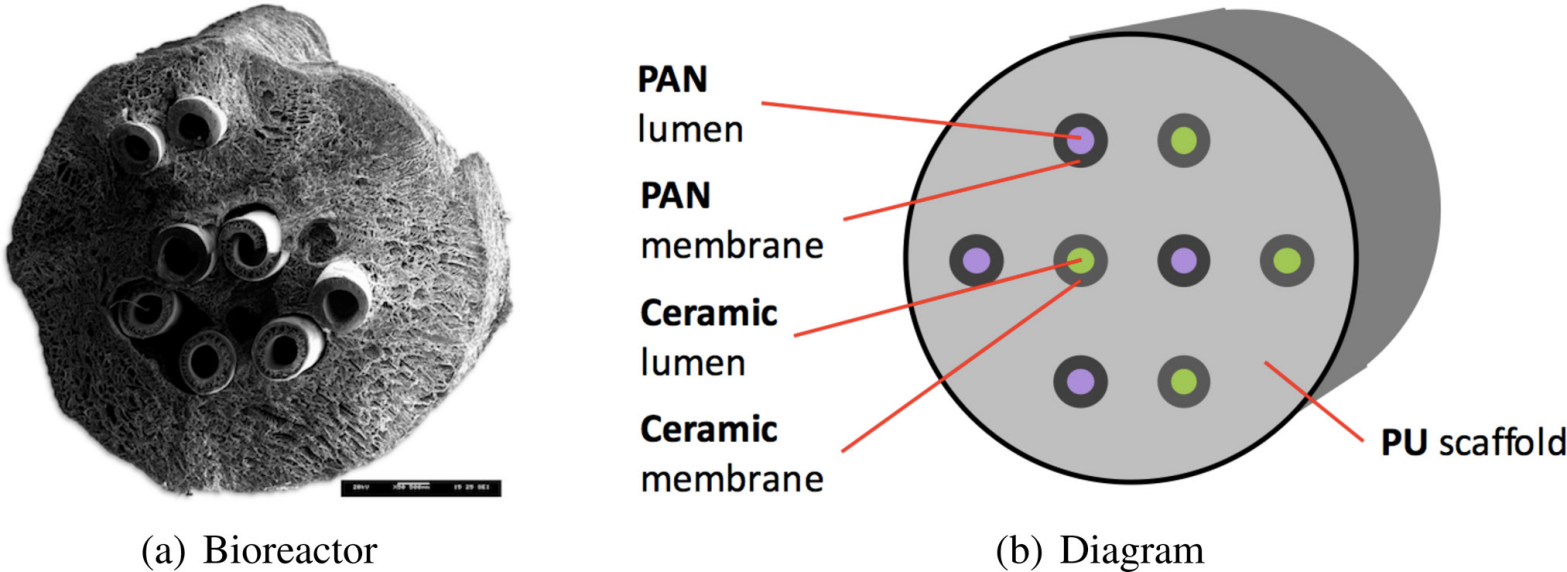
Dual hollow fiber bioreactor cross section. [Color figure can be viewed at http://wileyonlinelibrary.com]

As an example of the computational framework, we consider the RBC‐producing hollow fiber bioreactor illustrated in Figure 4. This novel, biomimetic, cost effective 3‐D hollow fiber bioreactor grows healthy blood *ex vivo*.^14^, ^15^ This bioreactor recapitulates the architectural and functional properties of blood formation and thereby reduces the need for growth factors (GFs) by an order of magnitude.^14^, ^23^ Nutrients, GFs, and oxygen flow through the hollow fibers via Poiseuille flow and diffuse into the 3‐D polymeric scaffold; resulting reactions cause the cells to grow, proliferate, and differentiate. Products and byproducts are excess cells and waste which diffuse out of the scaffold and exit through the hollow fibers. The dual hollow fiber design illustrated in Figure 5 allows recycling the expensive GFs in one set of capillaries (B) while taking up nutrients and discarding waste metabolites in another capillary set (A).^15^ We previously proposed a nominal superstructure optimization model for designing and operating the bioreactor.^24^ Rigorous, deterministic global optimization designed the nominal superstructure by simultaneously considering: number of parallelized bioreactor, number and type of hollow fibers, size and aspect ratio, feed concentrations, and flow rate through the bioreactor.

This article designs a computational framework analyzing how uncertain stem cell bioreactor performance impacts technology marketability. Our work fits into a broader vision of applying Quality by Design (QbD) toward reliable, reproducible, cellular therapy applications.^13^, ^25^, ^26^ Specifically, we manage uncertainty by: robustifying bioreactor design so that each reactor is likely to work; analyzing the price of robustification; quantifying how uncertainty affects bridging scales from individual reactions to production; identifying which sources of uncertainty profoundly impact bioreactor variability; comparing the impact of using different mathematical models; exploring the potential product marketability. Our computational analysis feeds back into experiments by suggesting candidate parameters validated using quantitative image analysis.^27^

## Computational Methods

Our overall computational goal is studying uncertainty using a baseline model and analyzing which sources of uncertainty have the most impact. The outcome of this study is to understand the quantitative tradeoffs for entering the RBC production market using a particular technology. We use as an example a model of RBC production in a dual hollow fiber bioreactor.^24^ The following subsections describe the component methods of our framework: mathematical models of stem cell fate; Krogh modeling and computational fluid dynamics (CFD); single variate and multivariate sensitivity analysis; superstructure optimization; robust optimization; NPV market analysis. Figure 1 shows the constituent parts and Figure 2 illustrates how they fit into a cohesive framework.

### Mathematical models of progenitor cell fate

White‐box, dynamic models of stem cell growth, proliferation, and differentiation are frequently developed by biologists, engineers, and mathematicians.^28^, ^29^, ^30^, ^31^, ^32^, ^33^, ^34^, ^35^, ^36^ Roeder^32^ summarizes these models, which conceptualize bioreactor cellular processes, for example, via molecular regulators^34^ or via network structure and dynamics.^36^ When we developed our optimization model for the blood cell bioreactor,^24^ we chose an ordinary differential equation (ODE) model relating hematopoiesis to the availability of GF proteins^33^; the Ma et al.^33^ model augments earlier hematopoiesis models.^28^, ^29^, ^31^ Using a discretized version of the Ma et al.^33^ model allowed us to relate growth kinetics to GF, the most expensive process input.^24^ For uncertainty analysis, we compare it with a competing literature hematopoiesis model; the Lobato da Silva et al.^30^ model of hematopoiesis in 2‐D suspension culture effectively bounds the performance of the 3‐D bioreactor. The RBC bioreactor will perform intermediate to human hematopoiesis^31^ and 2‐D suspension culture.^30^

### Krogh modeling and computational fluid dynamics

**Figure 6:**
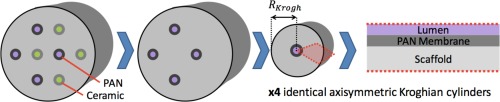
Krogh approximation applied to the dual hollow fiber bioreactor. [Color figure can be viewed at http://wileyonlinelibrary.com]

Misener et al.^24^ model mass transfer in the dual hollow fiber bioreactor following prior work.^37^, ^38^, ^39^, ^40^, ^41^, ^42^, ^43^, ^44^ Specifically, we use the Krogh^45^ approximation which models fluid flow within the bioreactor as an analytical function. But Krogh^45^ developed his original model, illustrated in Figure 6, with respect to oxygen distribution in muscle tissues where capillary densities range 379–2341 mm^−2^ 46; the assumption of evenly distributed, noninteracting capillaries may not be valid in a bioreactor which can accommodate 0.15–0.25 capillaries mm^−2^.^15^ The Krogh^45^ model validity is further challenged by: (1) extending the reactive species from O_2_ to additionally incorporate glucose, lactate, stem cell factor (SCF), and erythropoietin (EPO); and (2) the dual hollow fiber design of Macedo^14^ which allows selective species exchange such that adjacent hollow fiber have different characteristics. To quantify the error introduced by assuming the Krogh^45^ model, we use a high‐fidelity CFD model of mass transfer and numerically test the Krogh^45^ hypothesis using COMSOL 4.0a and finite element analysis. The *Computational Fluid Dynamics* section in the Supporting Information describes the complete setup that tests varying placements of hollow fibers so as to contradict the Kroghian assumption of equal spacing.

### Single variate and multivariate sensitivity analysis

Sensitivity analysis relates model parametric uncertainty to process design decisions and is therefore a commonly used tool in process engineering applications.^47^, ^48^, ^49^ Misener et al.^24^ use one‐at‐a‐time sensitivity analysis to test how parameter uncertainty affects optimization outcomes. But any variety of single‐parameter analysis makes it difficult to analyze the nonlinear parameter interactions common in biotechnology and bioengineering.^50^ Sensitivity analysis in optimization typically relates to shadow prices (Lagrangian multipliers) but, for the RBC bioreactor, we cannot use these sensitivities because: (1) the discrete design choices make the shadow prices meaningless; and (2) we want to study the parameters globally across their entire range.

Global sensitivity analysis (GSA), which estimates the effect of higher level indices across the entire parameter space,^51^ has been used in bioprocess engineering contexts including developing cell culture parameters.^52^ An alternative to GSA incorporates principal component analysis.^53^ It would be ideal to use GSA for optimizing the hematopoietic cell bioreactor, but the Misener et al.^24^ model has 30 possible parameters and each optimization run requires ≈300 s (mathematical optimization is typically more computationally demanding than mathematical simulation because each optimization may require many simulations or function calls). There are 435 combinations of two parameters ( 30·29/2) where, if we consider four levels of each parameter will take $\frac{30\cdot 29}{2}\cdot 4^{2}\cdot 300\;s=24.2$ CPU days; 4060 combinations of three parameters (902.2 CPU days); 27,405 combinations of four parameters (24,360 CPU days).

To mitigate, single variable and multivariable sensitivities analyze which parameter combinations have the most significant joint effect. Our strategy is similar to the Morris^54^ *elementary effects method* which copes with computationally expensive simulation models, but note that moving from a simulation to an optimization model implies considering optimization solution outputs rather than simulation sensitivities. For a linear optimization model, we would expect the sensitivity $S_{i,\;j}$ to be approximately 0; significantly large values of $S_{i,\;j}$ indicate that nonlinearity in the optimization model is affecting the final outcome.

### Global topological superstructure optimization

Typical superstructure optimization applications are in industry; examples include: well scheduling in petroleum fields,^55^, ^56^ crude oil scheduling in a petrochemical refinery,^57^ designing wastewater treatment systems,^58^, ^59^, ^60^ and crystallization.^61^ The purpose of Misener et al.^24^ applying superstructure optimization to a stem cell bioreactor is simultaneously testing a range of design variables leading to different superstructure possibilities; the ANTIGONE algorithm resolves design choice tradeoffs.^62^

The potential downside of superstructure optimization is that it is an expensive algorithm; deterministic global optimization involves divide‐and‐conquer search. The Misener et al.^24^ model therefore uses model sizes involving ≈200 variables and equations to achieve the reasonable 300 CPU seconds time performance noted in the single variate and multivariate sensitivity analysis section; limiting the number of parameters is also helpful in quantifying parametric uncertainty.

### Robust optimization

**Figure 7:**
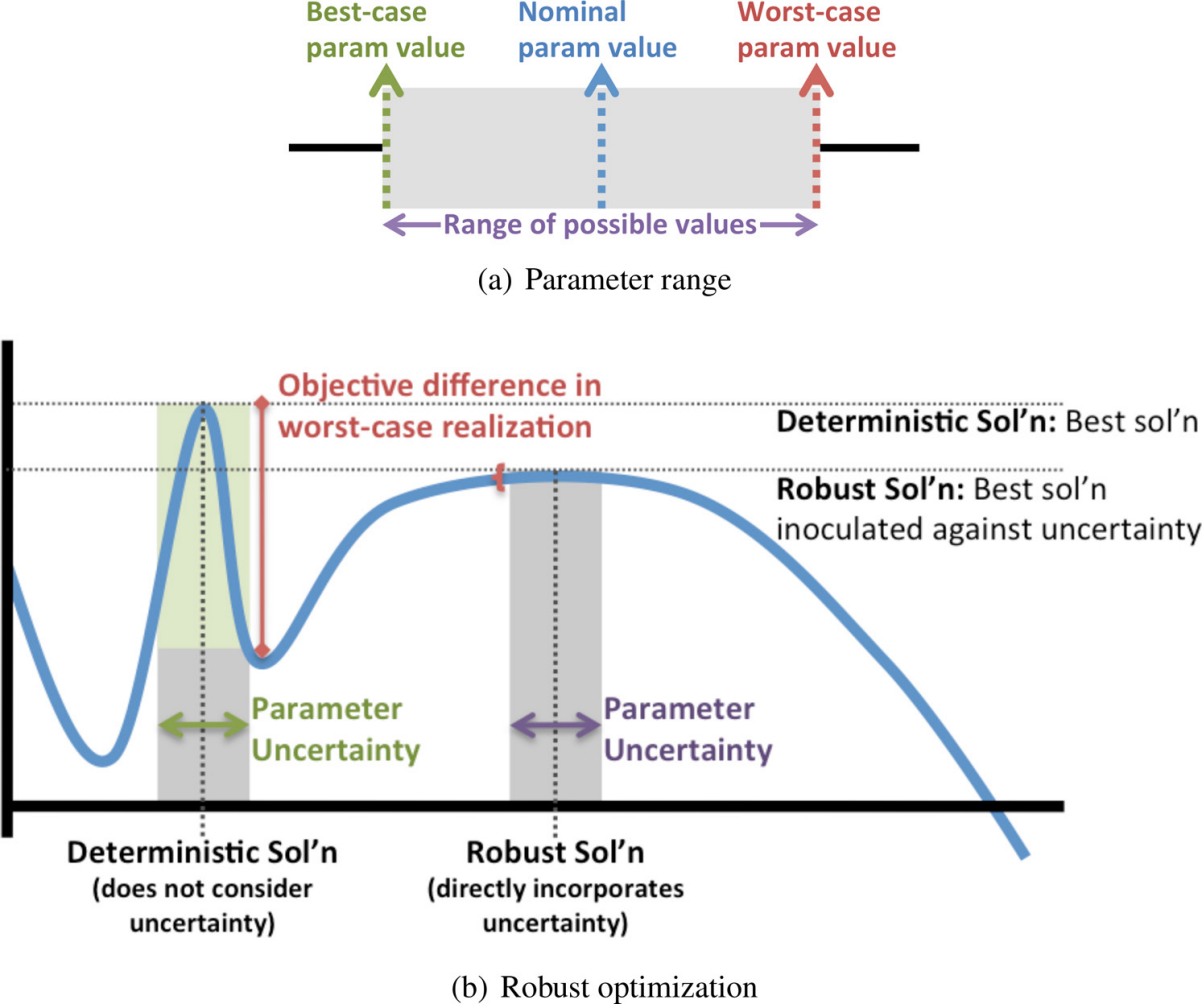
Robust optimization finds the best solution inoculated against parametric uncertainty; here we use it to produce a proactively defensive bioreactor design that takes into account the possibility of parameter variation. Optimality is guaranteed in the worst‐case parameter realization (a) and performance is also guaranteed to improve for the case of a continuum of scenarios (b). [Color figure can be viewed at http://wileyonlinelibrary.com]

Robust optimization, a strategy illustrated in Figure 7, finds the best solution inoculated against uncertainty; it is typically applied to instances of *strict uncertainty* where we can anticipate a range of possible parametric outcomes but not a probability distribution.^63^, ^64^, ^65^ Optimality is guaranteed in the worst‐case parameter realization and performance is also guaranteed to improve for the case of a continuum of scenarios.^66^

Strict uncertainty is most applicable to the RBC‐producing bioreactor because we have no knowledge of a probability distribution for any of the parameters except for the bioreactor material properties; see the analysis in Eq. (2) of Misener et al.^24^ Beyond assuming a uniform distribution for the parameter outcome, it is possible (with more experimental data) to permit a predetermined probability that a constraint can be violated; this prevents the recommended result from being too conservative. For now we focus on worst‐case analysis.

Robust optimization is a conservative strategy making the system more likely to operate within specifications; robust optimization allows proactively defensive bioreactor design. One possible problem with optimization is achieving solutions that are unrealistic because optimization pushes the system to the very best possible nominal solution. In practical application this is an issue because the “global optimum” may be highly sensitive to small parameter variation (see Figure 7). If the parameter realization is different than expected, the “optimal” solution with nominal parameters may perform very badly in the actual parameter realization. Robust optimization takes into account the possibility of parameter variation during the optimization process itself; this yields the best solutions that are inoculated against uncertainty.

### Net present value market analysis

NPV analyzes cash flows with respect to the time value of money; each cash inflow/outflow is discounted to its present value and the present values are summed

$$\text{NPV}(i,\;N)=\sum_{t=0}^{N}\frac{R_{t}}{{\left(1+i\right)}^{t}}$$

where $\left(t,\;R_{t}\right)$ pairs correspond to the time *t* and amount *R_t_* of each cash flow and *i* is the discount rate. We compare the cost inflows and outflows with respect to the expected: technology development costs, success probability, revenue stream, and market share.

## Mathematical Modeling Aspects

This section discusses mathematical modeling aspects dealing with introducing an alternative model for erythropoiesis, that is, RBC production, and a robust optimization framework. The nominal optimization model is fully characterized elsewhere^24^; the Supporting Information defines the optimization problem and all relevant symbols in Table S3.

### Ordinary differential equation models of hematopoiesis

**Figure 8:**
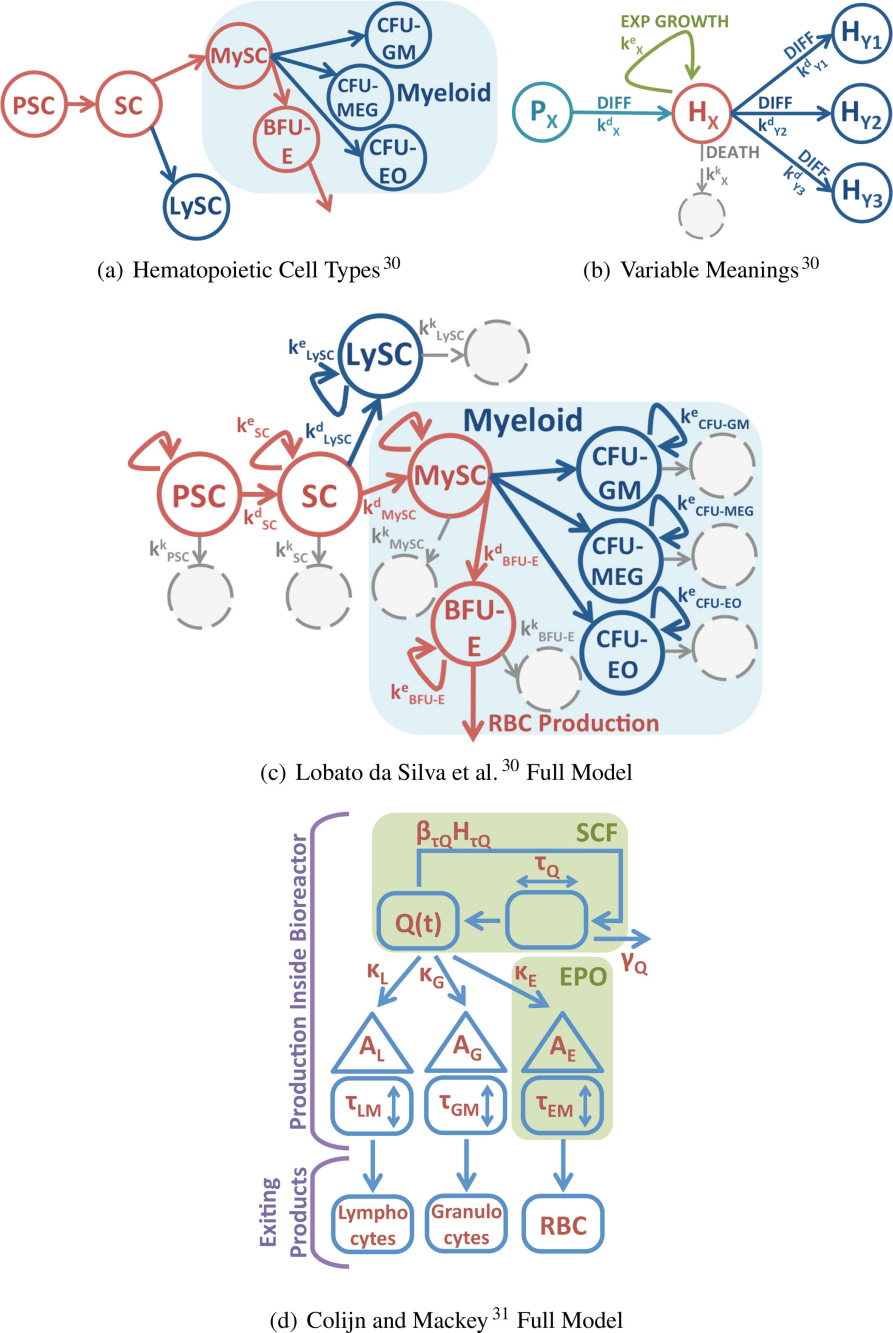
**The Lobato da Silva et al.** 30 **alternative model of hematopoiesis (illustrated in subfigures a–c) allows us to evaluate the possible error due to selecting the baseline Colijn and Mackey** 31 **model 2.** [Color figure can be viewed at http://wileyonlinelibrary.com]

The Colijn and Mackey^31^ model for hematopoiesis is the baseline for our optimal design^24^; an alternative model for cellular growth, proliferation, and differentiation was defined by Lobato da Silva et al.^30^ Figure 8 illustrates both our baseline and alternative models.

The cell types in Supporting Information Eq. (1) do not match the cell types defined by our baseline Colijn and Mackey^31^ model; both sets of authors take heterogeneous cell types from hematopoiesis and segment the cells into specific, definable types. We have already shown^24^ how to map the Colijn and Mackey^31^ cell types onto parameters defined by Chow et al.^67^ and Basford et al.^68^; these parameters connect the cellular growth, proliferation, and differentiation to the initialization and mass transfer portions of the model. Supporting Information Table S2 shows the new initialization and oxygen consumption parameters corresponding to the cellular definitions of Lobato da Silva et al.^30^

### Robust modeling counterpart

Supporting Information develops a robust counterpart for nominal oxygen consumption and designs equivalent robust counterpart models for each of the other uncertain parameters (see Supporting Information Table S4). This discussion significantly expands a conference paper^69^ where we used robust optimization on the bioreactor model but did not specify the mathematics.

## Results

Misener et al.^24^ justified a baseline mathematical model of the RBC‐producing bioreactor; this model is repeated in the Supporting Information. Supporting Information Table S3 defines the model indices, parameters, and variables. For the baseline mathematical model, we found a deterministic global optimum of $277 per unit of RBC (339 reactors; $0.82/reactor) where the material cost per reactor (after being lowered by massive parallelization) is driven by half‐life decay of EPO ($0.298) and SCF ($0.512). These bioreactor results compare favorably to the $8330 needed for 2‐D static culture.^70^

The following sections analyze the solution robustness via single variate and multivariate parameter sensitivity analysis, CFD modeling and exchanging models of hematopoiesis, and robust optimization. We show that, for the RBC‐producing bioreactor, the parameters interacting the most nonlinearly are those affecting the very smallest bioreactor scales. From a model uncertainty perspective, we show that and alternative hollow fiber placement and an alternative hematopoiesis model can be reformulated as parameter uncertainty and a 10% cost increase, respectively. Then, we directly use optimization under uncertainty to determine a more robust bioreactor configuration. Finally, we use our analysis to consider the potential for the RBC‐producing bioreactor to be a disruptive technology.

### Single variate and multivariate parameter sensitivity analysis

The baseline results are based partially on assumptions of parameters having specific values.^24^ For example, we assume that glucose uptake and lactate production are proportional to our experimental results^14^ and that every type of cell consumes metabolites/nutrients and produces waste in the same way. But Collins et al.^71^ show that nutrient consumption levels depend on the percentages of colony‐forming cells (CFU‐GM and BFU‐E); there is similar uncertainty in other parameters. As described in the *Computational Methods* section, we vary the 30 uncertain parameters over the Supporting Information Table S4 uncertainty ranges. Parameters with known error bars vary within their expected uncertainty levels; the remaining parameters were allowed to take values 50% (L1), 90% (L2), 110% (U1), and 150% (U2) of their nominal values.

Of the 30 parameters, 9 induced the global optimum to vary by 10% or more; these are presented in Supporting Information Table S5. The cellular flux uncertainty ${\hat{J}}_{\text{Cells}}$ induces the most variability; this is in line with our experimental observations that cells are being impeded from pushing through the ceramic hollow fibers of the bioreactor.^72^, ^73^ Next we investigate bivariate sensitivity; jointly changing the values of two parameters may have a nonlinear effect because the optimization model itself is nonlinear.

We see an interesting effect in Supporting Information Table S6 of bivariate sensitivities $S_{i,\;j}$: 13 of the 24 entries contributing to inducing >15% change are related to cellular kinetics and an additional 7 parameter entries are related to cellular consumption of nutrients and production of waste. These results are interesting because they imply that the parameters interacting the most nonlinearly in the model are those affecting the very smallest scales in the bioreactor. Note that it is common to observe that very small length and time scales in a simulation model may profoundly affect the largest length and time scales,^74^, ^75^ but this effect is more rare in engineering optimization models. This interaction of many length and time scales is one of the ways that superstructure optimization and its associated sensitivity analysis have to change from its original applications in industrial petrochemical systems toward tissue engineering.

### Exchanging models of mass transfer and hematopoiesis

**Figure 9:**
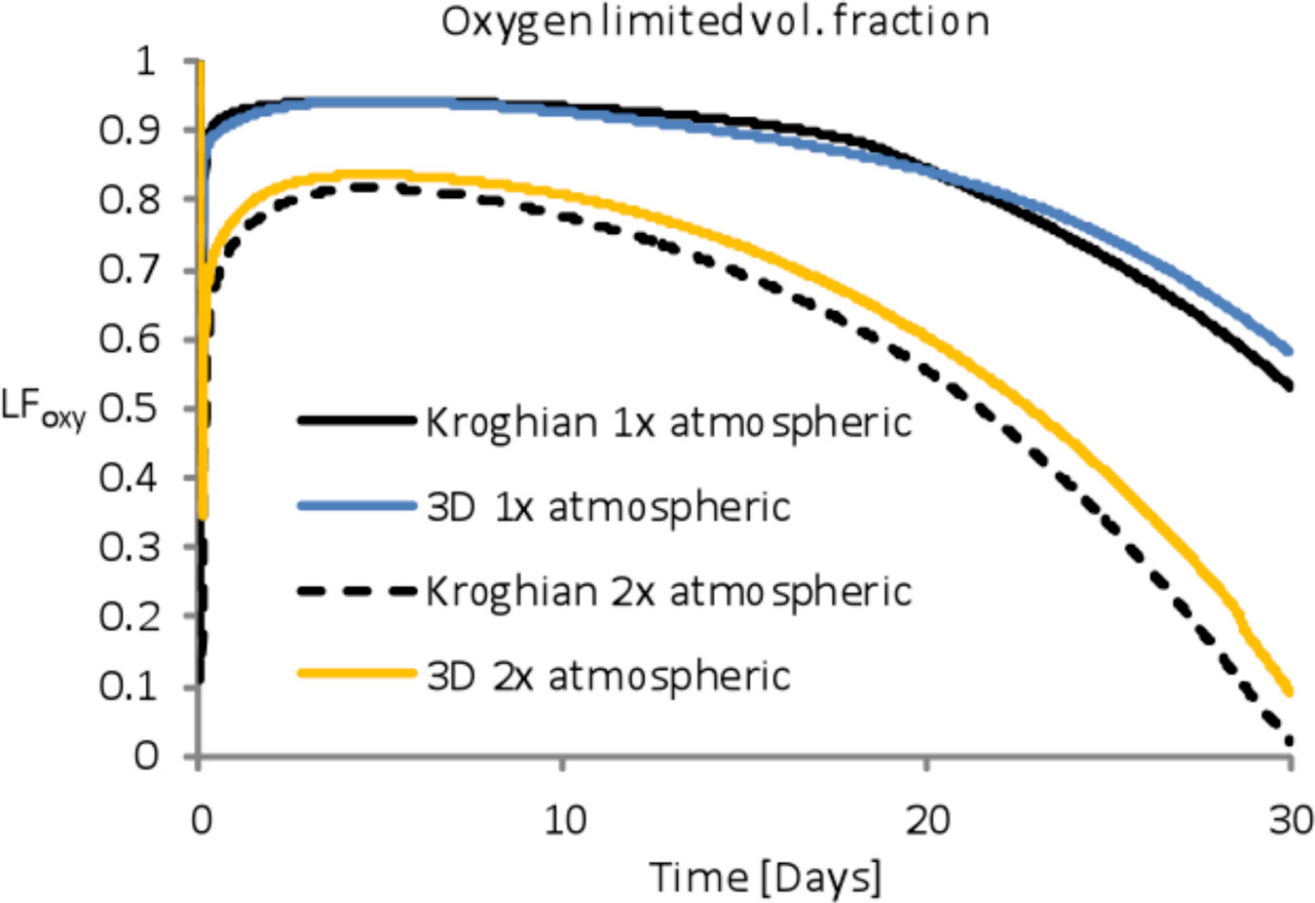
Comparison between the Krogh approximation and CFD Results. *Atmospheric* refers to O_2_ levels and *2x atmospheric* refers to a bioreactor with artificially increased O_2_ levels. This is only one run with evenly spaced hollow fibers. Note that there is more variability between different hollow fiber configurations, for example, Supporting Information Figure S13, than between the Krogh and CFD results. [Color figure can be viewed at http://wileyonlinelibrary.com]

The *Computational Fluid Dynamics* section in the Supporting Information describes how we set up the CFD analysis; Figure 9 and Supporting Information Figure S13 show results. The purpose of the CFD is to stress‐test the analytical Krogh^45^ approximation with respect to fluid flow within the experimentally observed bioreactors. Figure 9, which diagrams the bioreactor fraction limited by O_2_, indicates that, at least for one possible hollow fiber configuration, the Krogh and 3‐D CFD models have similar performance. But, at this writing, we have little control over bioreactor hollow fiber placement and do not know the hollow fiber placement until the bioreactor is cut open at the completion of an experiment. Supporting Information Figure S13 indicate that the variability induced by uncertainty in hollow fiber placement is more significant than the Kroghian vs. 3‐D CFD models. Based on these results, the Krogh approximation is well suited for this application; we assume that the parameter sensitivity analysis incorporates uncertainty due to hollow fiber placement.

In addition to switching between Kroghian and CFD models, we also considered replacing the hematopoiesis model of Colijn and Mackey^31^ with the model of Lobato da Silva et al.^30^; this change moved the price point on the reactor from $277 per unit of RBC to $306 per unit of RBC (a roughly 10% increase) but otherwise left the major design decisions the same from the ones we previously reported.^24^ The reason the design decisions stay constant is that, although the cell growth model has changed and the cells produce fewer RBC, the basic cellular need for nutrient delivery and waste clearance has not changed. This 10% cost increase with respect to cells yielded bounds the effect of using the wrong hematopoiesis model to describe the system; the Colijn and Mackey^31^ model of normal human hematopoiesis is probably too optimistic for an *ex vivo* system whereas the Lobato da Silva et al.^30^ 2‐D culture model may be too pessimistic for the biomimetic bioreactor.

### Robust optimization

Deterministic global optimization evaluated tradeoffs between multiple design parameters over multiple operating phases^24^; prior work had only optimized hollow fiber bioreactors over single variables under steady state assumptions.^38^, ^42^, ^43^, ^44^ But the nominal model is subject to uncertainty not only in the parameters but also in the model formulation itself; this uncertainty is the focus of the current article. Our quantitative image analysis confirms that key sources of uncertainty include^27^:

- **Variable distribution of hollow fibers**; the Krogh^45^ approximation of evenly distributed, noninteracting hollow fibers may not be valid for the RBC producing bioreactor.
- **Uncertain mass‐transfer coefficients** including: species diffusivities; hollow fiber and polyurethane scaffold porosities and pore structures; cellular flux leaving the scaffold.
- **Species reaction rates** of the five representative species (Glc, Lac, O_2_, EPO, and SCF) by each of the four representative cell types (hematopoietic stem cell, Gran, Leuk, and RBC).
- **Price and half‐life decay of GFs.**
- **Cellular growth, proliferation, differentiation**; parameters and the underlying model.

Using robust optimization, the nominal price for a unit of RBC increases from $277 to $383. This is a worst case estimate based on every one of the parameters taking the worst case realization with respect to Supporting Information Table S4; more bioreactor data would help us design more reasonable uncertainty rules which would allow moderate increases in the price while still handling the uncertainty in a reasonable way. But even before we have complete data on how the robust optimization parameters can be modified, it is important to note the way in which robust optimization proposes to change the bioreactor model. As expected, the robust framework recommends more reactors and GFs, but robust optimization also recommends a different configuration of polymeric and ceramic hollow fibers than the nominal optimization problem. Specifically, the hollow fiber type and number are limiting constraints robust framework recommends increasing the number of ceramic hollow fibers and decreasing the number of polymeric hollow fibers. The robust model assumes that fewer cells are likely to grow in the robust reactor but that more of the produced RBC is likely to exit the hollow fiber.

### Analyzing the potential to be disruptive technology

We previously estimated the bioreactor material cost,^24^ but we have thus far neglected the time‐value of money. To design an NPV market analysis, we assume:

- Our aim is to create a disruptive technology^19^, ^20^; therefore we only have 2 years to get a fully operational product.
- Based on Table 1 and the experimental Gantt charts in Figure 10: one operator can create and culture two bioreactors in parallel every 48 days and each operator can manage seven new experiments per year in duplicate.
  **Table 1:** Labor Time

Timeline		Days	Hours/Day	Person/Days	
Fiber creation	Fiber milling/extrusion	5	5	3.125	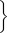 Sequential Steps
	Fiber sintering	4	1	0.5	
	Total	9		3.625	
Bioreactor fabrication	Fiber potting	1	4	0.5	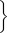 Sequential Steps
	Scaffold formation	11	1	1.375	
	Total	12		1.875	
Precondition and culture	Coating	3	4	1.5	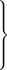 Sequential Steps
	Sterilization	1	11	1.375	
	Conditioning	3	0.5	0.1875	
	Seeding	1	14	1.75	
	Culture	35	2	8.75	
	Total	43		13.5625	
Terminal analyses		5	6	3.75	
Total time expenditure		69		22.8125
  **Figure 10:**
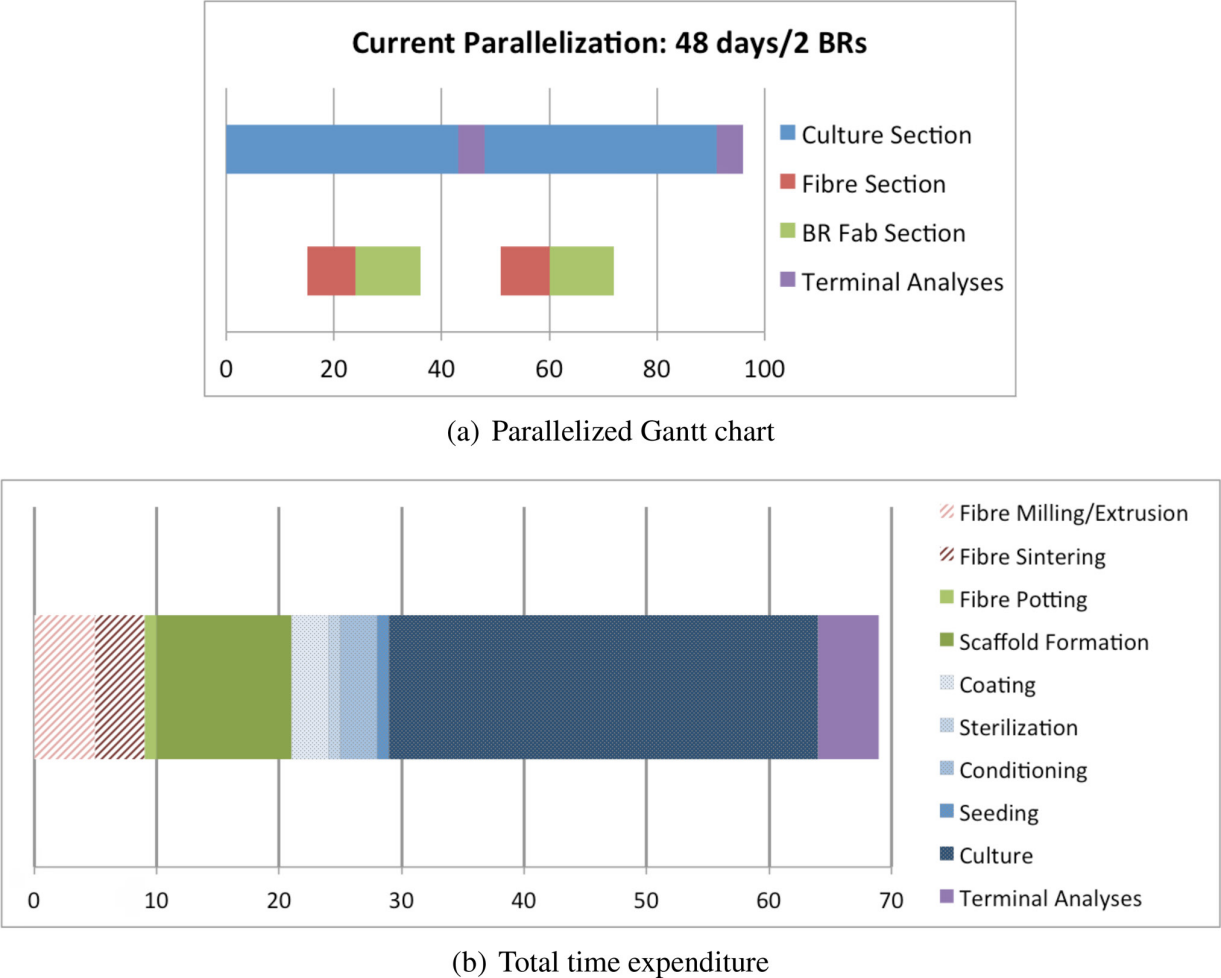
Labor time for fabricating the dual hollow fiber bioreactors. [Color figure can be viewed at http://wileyonlinelibrary.com]
- All material properties have been rigorously characterized.
- Species concentrations measurements are accurate both into and out of the reactor.
- We can quantitatively analyze each reactor at the end of an experiment, for example, using image analysis on bioreactor cross sections.^27^

We consider many types of uncertainty and design decisions, but assumptions (i)–(v) imply that all uncertainty can be handled by robust optimization except for uncertainty associated with the cells and their growth/proliferation/differentiation. So there are really only five design decisions which can induce unexpected changes: (1) bioreactor seeding density and (2–5) concentrations of O_2_, SCF, EPO, and glucose entering the bioreactor.

Based on the ODE model systems,^30^, ^33^ the cell kinetics functions are nonlinear. We aim to build a surrogate function using response surface methods^76^; this surrogate function may have similarities to the white‐box ODE hematopoiesis models,^30^, ^31^ but we cannot guarantee the form for either model and therefore the experiments will build a surrogate model over time. We expect that building a 5‐D response curve which is also informed by the ODE models mentioned previously will take on the order of 30, for example, $\approx 2^{5}$ if we consider a corner point design, experiments if we assume that the response is no more than quadratic. Therefore, in the first year, the project should employ four people to complete a total of 28 experiments; this will fully characterize the space. After that, the same four people will do 28 more experiments to exploit areas around the optimum a bit better.

But there is no reason to do experiments when the bioreactor is *not* going to be transformative. We consider several scenarios. The first is that we can construct the bioreactor in the price range of rare blood, $1150 to $3025 per unit RBC^70^; we take the average number of units rare blood requested each year in the United States of America (1800) and posit: (1) if there was greater rare blood availability, then it would be used more frequently and (2) that the proportion of rare blood used in the First World is proportional to that used in the United States of America. Therefore, we multiply the current rare blood usage, 1800 units per year in the United States of America by five to incorporate better availability and scale by the First World population ( $2.0\times 10^{9}/3.2\times 10^{8}=6.25$) to account for total usage; we conclude that the rare blood market share is $5.625\times 10^{4}$ units per year. We also posit that the total market share for blood shortages at storage banks and hospitals is a fraction of the total market share for blood and corresponds to the 10.3% of hospitals which experience at least 1 day yearly when blood shortages cannot be met^70^; those data imply $9.2\times 10^{7}\cdot 0.103/365=2.60\times 10^{4}$ units of blood needed to cover yearly shortages. We also estimate that the military would be willing to pay for $5.0\times 10^{4}$ units of blood per year for security and defense. We therefore estimate that the entire market share of rare blood is $1.32\times 10^{5}$ units per year; note that this an underestimate of the rare blood needed since it excludes Africa where there are significantly higher populations of rare blood types and rare blood diseases.^77^, ^78^ Assuming a 11% discount rate for NPV^79^ and a 10.4% chance success chance of clearing clinical trials,^80^ we say that for the first two years our costs will scale with the four parallel operators; we assume that each person plus lab and material costs will be $150k per year so that in the first two years we will pay out $600k per year. In years 3–20, we assume that we have 40% market share.^81^ In total, the payout is (using NPV) $\$1.16\times 10^{8}$ which does not compensate for the expected $\$1.098\times 10^{9}$ prehuman capitalized cost of an average compound plus the $\$1.460\times 10^{9}$ clinical cost of the average compound.

But if we could compete with the current human transfusion market and price the product at $225, then we could expect that the total market share would be $\$9.2\times 10^{7}$ per year. Using the same assumption of $\$6\times 10^{5}$ per year for 2 years, and 40% market saturation in year 3 and thereafter,^81^ the revenue would be $\$6.08\times 10^{9}$ over 20 years; this is enough to clear regulatory approval in both prehuman and clinical trials. If we have miscalculated the discount rate by 10%, then the revenue could be as little as $\$4.79\times 10^{9}$ or as much as $\$7.17\times 10^{9}$ over 20 years.

Our current estimate for robustified bioreactor design is that the bioreactor is priced at $383 in the worst case; we assume that storage and delivery is a further $225 (since this is the storage and delivery price for the already‐free blood) and assume that the final blood price would be $800; at that price we can beat the rare blood market by a significant fraction and therefore the market share is an order of magnitude larger than for rare blood. Then the payout would be $\$3.096\times 10^{8}$ which is still not enough to clear regulatory. We estimate that the price point becomes more reasonable when we are able to charge $500 and we therefore estimate that at that point the blood product could cover 20% of the total market since people may be willing to pay more for the ease‐of use. Then the payout would be $\$2.7\times 10^{9}$ over the 20 years and therefore worthwhile. To get down to $500, we need several things in the bioreactor to be better. First, the cell flux through the ceramic hollow fibers needs to be better; to manage this is basically a material question to ask whether it is possible to have hollow fibers through which the cells can migrate without shear‐related damage. With better production rates through the membrane, we expect that this could be a disruptive technology; the disruptive potential of the technology radically increases if this RBC production technology significantly cuts down on the current 1% immunologic reaction rate for RBC transfusions.^82^ Also, the recently developed immortalized adult human erythroid line^12^ may help the bioreactor by allowing production runs longer than 30 days.

## Discussion

Although the nominal optimization model indicated that we *could* design a bioreactor costing roughly $277 per unit of RBC,^24^ the model did not incorporate uncertainty and therefore we had no way of evaluating if our design decisions would stand up to scrutiny. Based on this analysis, we find that, for this particular bioreactor, there are a number of parameters including cellular flux, species half‐life, and cellular kinetic parameters which should be analyzed in greater detail to find the exact price point of the bioreactor. For this hematopoietic cell bioreactor, the robust framework recommends hedging against uncertainty with more reactors and GFs. The robust framework recommends increasing the number of ceramic hollow fibers and decreasing the number of polymeric hollow fibers with respect to the nominal optimum. We also note that robust optimization is a useful framework for explicitly incorporating the parameter uncertainty into the optimization model; we can defensively design the bioreactor to accommodate known parameter variability.

There are several novel items here. First, neither superstructure optimization nor robust optimization have been applied to stem cell tissue engineering; our proposed computational framework adapts techniques which have proved successful in well‐established industries and transforming these strategies to optimize stem cell bioprocesses. One significant way that superstructure optimization changes from heavy industries to stem cell bioprocessing is the need to incorporate more orders of magnitude into the constraint set. In examples such as water treatment, there will be small quantities such as trace metals and small amounts of sulfur, but these may be linearly scaled such that the condition number of the constraint matrix is small, that is, the problem is well scaled. The superstructure optimization problem for stem cell bioprocessing, however, involves many orders of magnitude ranging from the individual cellular reactions to the number of cells produced. The complexity here is the interaction between different scales.

For robust optimization, we quantitatively ask how much risk is acceptable. In petrochemical process optimization, for example, engineers evaluate how close they are willing to get to environmental constraints; violating the law may result in a fine while staying too conservative may reduce product margins. Moving toward a clinical application, the risk incurred is that the process does not make sufficient blood for a patient in need. On the other hand, acting very conservatively may increase the price dramatically; robust optimization weighs the risk between not providing a necessary product and making it too expensively.

Combining many different uncertainty analysis strategies into one model is especially powerful; stem cell bioprocessing does not admit certain models or certain parameters. Modeling and optimization quantitatively characterize a system with minimal experiments, but we cannot tie ourselves to a single uncertainty analysis method; we want to build consensus between disparate analysis types. The different framework components reinforce one another; the computational techniques illustrated in Figure 1 are combined using the Figure 2 recipe.

## Conclusions

This article proposes a framework for stem cell bioreactor design under uncertainty and analyzes the associated mathematical tool kit. In stem cell biomanufacturing, an optimal but nonrobust design may be sensitive to model or parametric uncertainty. Our framework directly incorporates uncertainty considerations and therefore fits into the broader vision of QbD.

## Supporting information

Supporting Information 1
