# Supplementary material for: Stem cell biomanufacturing under uncertainty: A case study in optimizing red blood cell production
Source: AIChE J. 2017 Dec 7;64(8):3011–22. doi: 10.1002/aic.16042 (PMC6108044; doi:10.1002/aic.16042)
Supplement: Supplementary file 1 — Supporting Information 1 [file AIC-64-3011-s001.pdf]

Supplementary Material

Stem Cell Biomanufacturing under Uncertainty:
A Case Study in Optimizing Red Blood Cell Production

Misener • Allenby • Fuentes Garí • Gupta • Wiggins
Panoskaltsis • Pistikopoulos • Mantalaris

Computational Fluid Dynamics

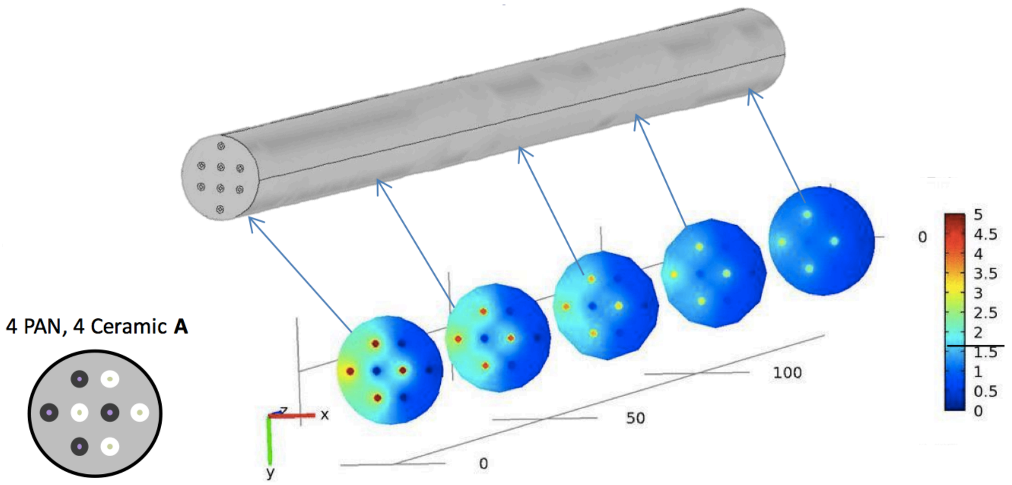

Figure 11: Example of Computational Fluid Dynamics Visualization

We designed a 3D bioreactor representation in the computational fluid dynamics (CFD) software COMSOL; Figure 11 diagrams an example system. We tested many possible geometries of hollow fiber including the examples illustrated in Figure 12 with 6, 8 , and 10 hollow fibers; we also tried varying configurations of 3 PAN / 3 CRM, 4 PAN / 4 CRM, and 5 PAN / 5 CRM hollow fibers (Figure 13). We designed our mesh with 9.37×10^3 elements (average quality: 0.238) and used a *direct* solver within COMSOL. The results in Figure 14 represent the bioreactor fraction which is glucose limited ($LF_{GLU} \in [0, 1]$) for the 30 days of the culture. Observe that more variability is induced via our inability to predict hollow fiber configuration *a priori* (Figure 14) than by the Krogh approximation (Figure 9).

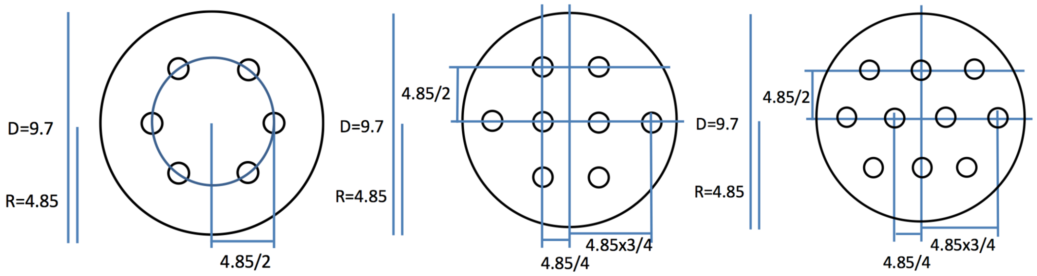

(a) Hollow fiber: 3 PAN; 3 CRM (b) Hollow fiber: 4 PAN; 4 CRM (c) Hollow fiber: 5 PAN; 5 CRM

Figure 12: CFD Geometry Specifications

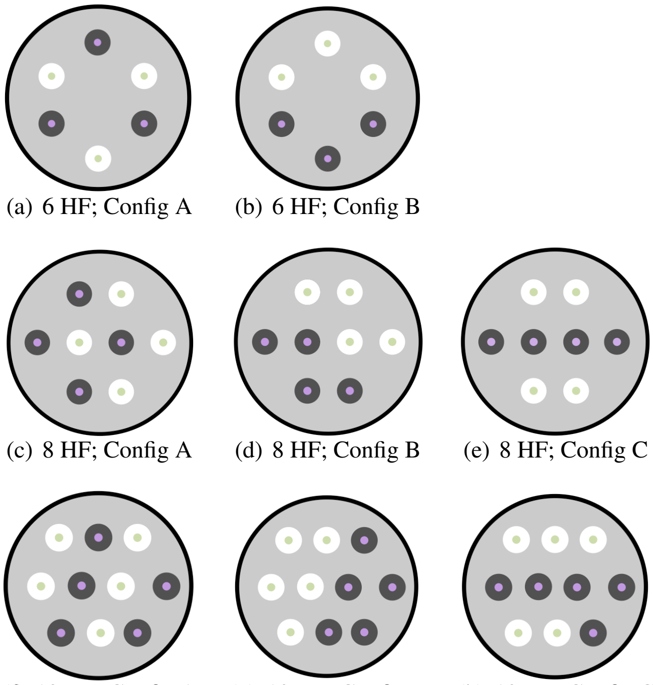

(a) 6 HF; Config A (b) 6 HF; Config B

(c) 8 HF; Config A (d) 8 HF; Config B (e) 8 HF; Config C

(f) 10 HF; Config A (g) 10 HF; Config B (h) 10 HF; Config C

Figure 13: Hollow fiber (HF) configurations (a) – (h) were used to initialize the simulations in Figure 14.

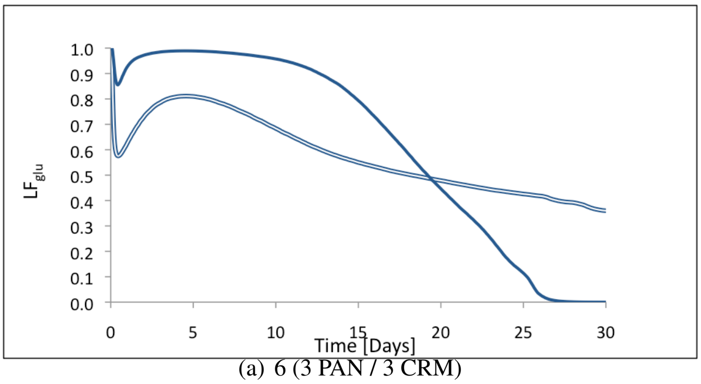

(a) 6 (3 PAN / 3 CRM)

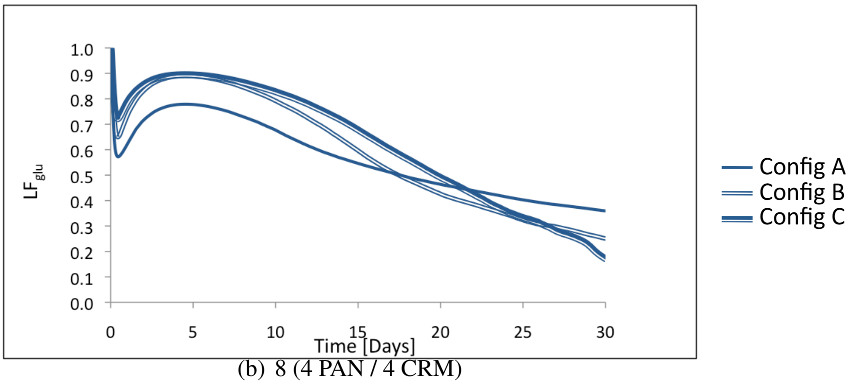

(b) 8 (4 PAN / 4 CRM)

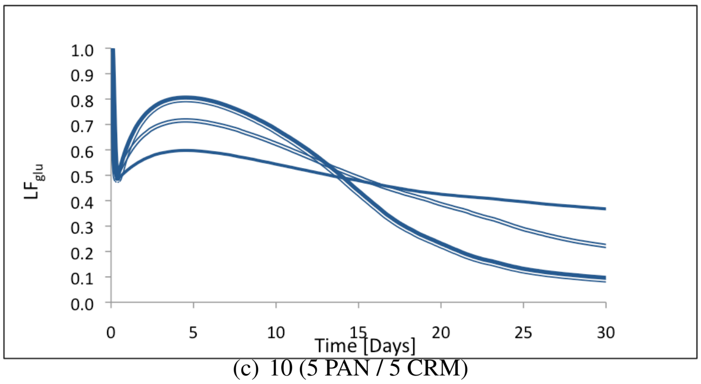

(c) 10 (5 PAN / 5 CRM)

Figure 14: Simulations representing the glucose-limited bioreactor fraction over a 30-day culture. These simulations were run using the geometry specifications in Figure 12 and the hollow fiber placements in Figure 13.

Baseline Optimization Problem

Misener et al.[24] introduce the full mathematical model describing this system; the following model summarizes that work and highlights in **red bold** the uncertain parameter values whose value is fixed at a baseline here. Recall that this model approximates the dynamic system by discretizing the ODEs into 5 weeks, $w \in \{1, \ldots, 5\}$.

$$\min \quad \left[\sum_{k\in\{\mathrm{EPO;\,SCF}\}} \mathbf{p_k}\cdot\tau_k\cdot N_{\mathrm{R}}\cdot \mathrm{Vol}_{\mathrm{Recyc}}\cdot C_{k,\mathrm{IN}}+\mathbf{p_k}\cdot D\cdot V_k\cdot \mathrm{Vol}_T\right]+\left[\sum_{h\in\{Q;\,E;\,G;\,L\}} p_{\mathrm{UCB}}\cdot H_{h,0}\cdot \mathrm{Vol}_T\right]$$

s.t.

Superstructure Topology

$$\begin{cases} N_{\mathrm{HF}} &= N_{\mathrm{HF,PAN}}+N_{\mathrm{HF,CRM}}\\ \varepsilon_{\mathrm{R,HF}}\cdot R_4^2 &\geq N_{\mathrm{HF,PAN}}\,R_{2,\mathrm{PAN}}^2+N_{\mathrm{HF,CRM}}\,R_{2,\mathrm{CRM}}^2\\ \mathrm{Vol}_R &= \pi\,(L-L_e)\,\left[R_4^2-N_{\mathrm{HF,PAN}}\,R_{2,\mathrm{PAN}}^2-N_{\mathrm{HF,CRM}}\,R_{2,\mathrm{CRM}}^2\right]\\ \mathrm{Vol}_{K,k} &= \begin{cases} \frac{\pi\cdot(L-L_e)\cdot R_4^2}{N_{\mathrm{HF,PAN}}} & k=\mathrm{Glc,\,Lac}\\ \frac{\pi\cdot(L-L_e)\cdot R_4^2}{N_{\mathrm{HF,CRM}}+N_{\mathrm{HF,PAN}}} & k=\mathrm{O_2}\\ \frac{\pi\cdot(L-L_e)\cdot R_4^2}{N_{\mathrm{HF,CRM}}} & k=\mathrm{EPO,\,SCF}\end{cases}\\ R_{3,k} &= \sqrt{\frac{\mathrm{Vol}_{K,k}}{\pi\,(L-L_e)}} \qquad \forall\,k\\ \mathrm{Vol}_T &= N_{\mathrm{R}}\,\mathrm{Vol}_{\mathrm{R}} \end{cases}$$

Production Requirement

$$\begin{cases} H_{Q,w}+H_{L,w}+H_{E,w}+H_{G,w} \leq H_{\mathrm{MAX}} & \forall\,w\in\{1,\ldots,5\}\\ \gamma_{P,w}\leq \frac{\mathbf{\hat{J}_{Cells}}}{\Delta\hat{H}_{\mathrm{Cells}}}\cdot\pi\cdot\frac{N_{\mathrm{HF,CRM}}\cdot R_{2,\mathrm{CRM}}\cdot L}{\mathrm{Vol}_R} & \forall\,w\in\{1,\ldots,5\}\\ \sum_{w>1}\gamma_{P,w}\,D_t\,\frac{H_{E,w}+H_{E,w-1}}{2} \geq \mathrm{Unit}_{\mathrm{RBC}} \end{cases}$$

Species Transfer

$$\begin{cases} C_{k,\mathrm{MIN}} \leq C_{k,i}(r,z)\leq C_{k,\mathrm{MAX}} & \forall\,k,i\\ C_{k,\mathrm{OUT}} = \left(R_2-\frac{R_3^2}{R_2}\right)\cdot\left(\frac{\mathrm{N}_{HF}\cdot V_k}{U_Z}\right)+\mathrm{C}_{k,\mathrm{IN}} & \forall\,k\\ C_{k,\mathrm{LOSS}} = \frac{V_k}{\mathbf{D_{k,3}}}\,\left(R_3^2-R_2^2\right)\,\left(\frac{11}{48}+\frac{1}{2\cdot\boldsymbol{\varepsilon}_{\mathbf{HF}}}\ln\frac{R_2}{R_1}\right) & \forall\,k\\ C_{k,3}(R_2,0) = C_{k,\mathrm{IN}}-C_{k,\mathrm{LOSS}} & \forall\,k\\ C_{k,3}(R_3,L) = \frac{V_k}{\mathbf{D_{k,3}}}\,\left(\frac{R_3^2}{2}\ln\frac{R_3}{R_2}-\frac{R_3^2-R_2^2}{4}\right)+C_{k,\mathrm{OUT}}-C_{k,\mathrm{LOSS}} & \forall\,k \end{cases}$$

Species Reaction

$$\begin{cases} V_k = \frac{\hat{U}_Z\cdot(\hat{C}_{k,\mathrm{IN}}-\mathbf{\hat{C}_{k,OUT}})}{\hat{L}} & \forall\,k\in\{\mathrm{Glc,\,Lac}\}\\ V_{O_2} = \mathbf{V_{O_2,Q}}\cdot H_{Q,w}+\mathbf{V_{O_2,L}}\cdot H_{L,w}+\mathbf{V_{O_2,E}}\cdot H_{E,w}+\mathbf{V_{O_2,G}}\cdot H_{G,w} \end{cases}$$

Half-Life Decay

$$\Big\{\ \tau_k = \lim_{n\to\infty} 2^n\,\frac{T}{\mathbf{t_{1/2}}}\,\left(1-2^{-1/2^n}\right) \qquad \forall\,k\in\{\mathrm{Glc,\,Lac}\}$$

Proliferation & Differentiation

$$\begin{cases} \frac{H_{Q,w}-H_{Q,w-1}}{\Delta t} = -\left(\kappa_{E,w-1}+\kappa_{G,w-1}+\kappa_{L,w-1}\right)\cdot H_{Q,w-1}\\ \qquad\qquad +\left(2\cdot e^{-\boldsymbol{\gamma}_{\mathbf{Q}}\cdot\tau_Q}-1\right)\cdot\beta_{Q,w-1}\cdot H_{Q,w-1}\\ \frac{H_{E,w}-H_{E,w-1}}{\Delta t} = -(\boldsymbol{\gamma}_{\mathbf{E}}+\gamma_{P,w})\cdot H_{E,w-1}+\mathbf{A_E}\cdot\kappa_{E,w-1}\cdot H_{Q,w-1}\\ \frac{H_{L,w}-H_{L,w-1}}{\Delta t} = -(\boldsymbol{\gamma}_{\mathbf{L}}+\gamma_{P,w})\cdot H_{L,w-1}+\mathbf{A_L}\cdot\kappa_{L,w-1}\cdot H_{Q,w-1}\\ \frac{H_{G,w}-H_{G,w-1}}{\Delta t} = -(\boldsymbol{\gamma}_{\mathbf{G}}+\gamma_{P,w})\cdot H_{G,w-1}+\mathbf{A_G}\cdot\kappa_{G,w-1}\cdot H_{Q,w-1}\\ \qquad\qquad w\in\{2,\ldots,5\} \end{cases}$$

Growth Rate Bounds

$$\begin{cases} \kappa_{E,w} \in [\boldsymbol{\kappa}^{\mathbf{LO}}_{\mathbf{E,w}},\,\boldsymbol{\kappa}^{\mathbf{UP}}_{\mathbf{E,w}}]\\ \kappa_{G,w} \in [\boldsymbol{\kappa}^{\mathbf{LO}}_{\mathbf{G,w}},\,\kappa^{\mathrm{UP}}_{G,w}]\\ \kappa_{L,w} \in [\boldsymbol{\kappa}^{\mathbf{LO}}_{\mathbf{L,w}},\,\kappa^{\mathrm{UP}}_{L,w}] \end{cases}$$

Our optimal design is based on the Colijn and Mackey[31] hematopoiesis model[24]; a discretized version is labeled *Proliferation & Differentiation* in the preceding model. But there are alternate models of hematopoiesis; we have no guarantee that the Colijn and Mackey[31] model is correct. The Lobato da Silva et al.[30] model for cellular growth, proliferation, and differentiation, illustrated in Figure 8, has the following form:

$$\begin{aligned}\frac{dH_h}{dt} =& k_h^e\, \gamma\, H_h + k_h^d\, [P_h] + \sum_Y k_Y^e\, H_h - k_h^k H_h \\ & \forall\, h \in \{\text{PSC, SC, MySC, LySC, CFU-GM, CFU-MEG, BFU-E, CFU-EO}\}\end{aligned} \tag{1}$$

where $H_{h,w}$ is the concentration of each cell type; $[P_h]$ is the concentration of the parent cell type; γ is a factor limiting expansion; k_h^e, k_h^d, and k_h^k represent expansion, differentiation, and death, respectively. Lobato da Silva et al.[30] further define a maturation map (illustrated in Figure 8) between the cell types: pluripotent stem cells (PSC) become stem cells (SC); SC become myeloid stem cells (MySC) or lymphoid stem cells (LySC); MySC become colony-forming unit - granulocyte, macrophage (CFU-GM), colony-forming unit - megakaryocyte (CFU-MEG), burst-forming unit - erythroid (BFU-E), or colony-forming unit - eosinophils (CFU-EO); BFU-E become colony-forming unit - erythroid (CFU-E). Figure 8 shows the path for driving stem cells towards becoming RBC precursor CFU-E[30].

To test the alternate model of hematopoiesis, we substitute the equations for maximum cell density (H_{MAX}) and the reaction rate (V_{O_2}) of O_2 with equivalent equations which are functions of the cell types in the Lobato da Silva et al.[30] model (PSC, SC, MySC, LySC, CFU-GM, CFU-MEG, BFU-E, CFU-EO); the new coefficients for the reaction rates ($V_{\text{O}_2,h}$) and initialization count with respect to each different cell type are taken from Table 2. Additionally, the following equations for the *Proliferation & Differentiation* equations in the baseline optimization problem change:

$$
\begin{aligned}
\frac{H_{\mathrm{PSC},w}-H_{\mathrm{PSC},w-1}}{\Delta t} &= k^e_{\mathrm{PSC}} \cdot \gamma_{w-1} \cdot H_{\mathrm{PSC},w-1} - k^d_{\mathrm{SC}} \cdot H_{\mathrm{PSC},w-1} - k^k_{\mathrm{PSC}} \cdot H_{\mathrm{PSC},w-1} \\
&\qquad w \in \{2,\ldots,5\} \\
\frac{H_{\mathrm{SC},w}-H_{\mathrm{SC},w-1}}{\Delta t} &= k^e_{\mathrm{SC}} \cdot \gamma_{w-1} \cdot H_{\mathrm{SC},w-1} + k^d_{\mathrm{SC}} \cdot H_{\mathrm{PSC},w-1} - \\
&\quad (k^d_{\mathrm{MySC}} + k^d_{\mathrm{LySC}} + k^k_{\mathrm{SC}}) \cdot H_{\mathrm{SC},w-1} \qquad w \in \{2,\ldots,5\} \\
\frac{H_{\mathrm{MySC},w}-H_{\mathrm{MySC},w-1}}{\Delta t} &= k^e_{\mathrm{MySC}} \cdot \gamma_{w-1} \cdot H_{\mathrm{MySC},w-1} + k^d_{\mathrm{MySC}} \cdot H_{\mathrm{SC},w-1} - \\
&\quad (k^d_{\text{CFU-GM}} + k^d_{\text{CFU-MEG}} + k^d_{\text{BFU-E}} + k^d_{\text{CFU-EO}} + k^k_{\mathrm{MySC}}) \cdot H_{\mathrm{MySC},w-1} \\
&\qquad w \in \{2,\ldots,5\} \\
\frac{H_{\mathrm{LySC},w}-H_{\mathrm{LySC},w-1}}{\Delta t} &= k^e_{\mathrm{LySC}} \cdot \gamma_{w-1} \cdot H_{\mathrm{LySC},w-1} + k^d_{\mathrm{LySC}} \cdot H_{\mathrm{SC},w-1} - k^k_{\mathrm{LySC}} \cdot H_{\mathrm{LySC},w-1} \\
&\qquad w \in \{2,\ldots,5\} \\
\frac{H_{h,w}-H_{h,w-1}}{\Delta t} &= k^e_h \cdot \gamma_{w-1} \cdot H_{h,w-1} + k^d_h \cdot H_{\mathrm{MySC},w-1} - k^k_h \cdot H_{h,w-1} \\
&\quad h \in \{\text{CFU-GM, CFU-MEG, BFU-E, CFU-EO}\}\,;\ w \in \{2,\ldots,5\}\,.
\end{aligned}
$$

Data, Definitions and Parameter Values

Table 2: Absolute number of cells after umbilical cord blood processing[68] and specific O_2 uptake rate parameters of cell types in the bone marrow hematopoietic compartment[67]

Abbrev	Cell Type (h)	Cell # ($\times 10^7$ cells)	Specific O_2 Uptake Rate ($V_{O_2,h}$, mol_{O_2}/cell/day)
PSC	Pluripotent Stem Cell	0.024	3.89×10^{-14}
SC	Stem Cell	3.580	3.89×10^{-14}
MySC	Myeloid Stem Cell	0.026	3.89×10^{-14}
LySC	Lymphoid Stem Cell	24.934	1.68×10^{-14}
CFU-GM	CFU, Granulocyte, Macrophage	30.085	1.56×10^{-11}
CFU-MEG	CFU, Megakaryocyte	–	3.46×10^{-11}
BFU-E	BFU, Erythroid	–	1.56×10^{-13}
CFU-E0	CFU, Eosinophils	–	1.56×10^{-11}

CFU: Colony Forming Unit; BFU: Burst Forming Unit

Table 3: Definitions

	Symbol	Description	Units	Source
Abbreviations	BM	Bone marrow		
	CFD	Computational fluid dynamics		
	GF	Growth factor		
	HF	Hollow fiber		
	HSC	Hematopoietic stem cell		
	MINLP	Mixed-Integer Nonlinear optimization Problem		
	ODE	Ordinary Differential Equation		
	PAN	Polyacrylonitrile		
	PU	Polyurethane		
	RBC	Erythrocyte; red blood cell		
	QbD	Quality by Design		
	UCB	Umbilical cord blood		
Indices	h	Hematopoietic cells; For the model of Colijn and Mackey[31]: $h \in \{Q, E, L, G\}$; $Q \equiv$ HSC, $E \equiv$ RBC, $L \equiv$ lymphocytes, $G \equiv$ granulocytes; For the model of Lobato da Silva et al.[30]: $h \in \{$PSC, SC, MySC, LySC, CFU-GM, CFU-MEG, BFU-E, CFU-EO, CFU-E$\}$; PSC $\equiv$ pluripotent stem cells, SC $\equiv$ stem cells, MySC $\equiv$ myeloid stem cells, LySC $\equiv$ lymphoid stem cells, CFU-GM $\equiv$ colony-forming unit - granulocyte, macrophage, CFU-MEG $\equiv$ CFU - megakaryocyte, BFU-E $\equiv$ burst-forming unit - erythroid, CFU-E_0 $\equiv$ CFU - eosinophils, CFU-E $\equiv$ CFU - erythroid		
	i	Bioreactor region; $i \in \{1, \ldots, I\}$; $1 \equiv$ Hollow fiber lumen, $2 \equiv$ Hollow fiber membrane, $3 \equiv$ polyurethane scaffold; one Krogh cylinder, $4 \equiv$ entire Bioreactor		
	k	Species; $k \in \{$Glc, Lac, O_2, EPO, SCF$\}$; Glc $\equiv$ glucose, Lac $\equiv$ lactate, $O_2 \equiv$ oxygen, EPO $\equiv$ erythropoietin, SCF $\equiv$ stem cell factor		
	t	Time period in weeks; $w \in \{1, \ldots, 5\}$		
Parameters	$\varepsilon_{R,HF}$	Maximum HF packing density in reactor	0.14	Macedo[14]
	D	Days at steady state culture conditions	35 days	Macedo[14]

continued on the next page

Parameters

Table 3 (Definitions) continued

	Symbol	Description	Units	Source
	$\Delta\hat{H}_{\text{Cells}}$	Experimental cellular concentration across membrane	1.00×10^6 cells / mm^3	Macedo [14]
	R_i	Radius of region $i \in \{1, 2\}$	$R_{\text{CRM},1} = 0.25$ mm; $R_{\text{CRM},2} = 0.43$ mm	Macedo [14]
			$R_{\text{PAN},1} = 0.26$ mm; $R_{\text{PAN},2} = 0.45$ mm	Macedo [14]
	Unit_{RBC}	Number of RBC in one unit	2×10^{12} cells	Timmins and Nielsen [70]
	$\text{Vol}_{\text{Recyc}}$	Recycle volume needed for each reactor	1.0×10^{-4} m^3	est.
Uncertain Parameters	A_h	Amplification parameter for differentiation $h \in \{E, L, G\}$	$A_E = 5.63 \times 10^5$; $A_G = 2.82 \times 10^5$; $A_L = 7.52 \times 10^4$	Colijn and Mackey [31]
	$C_{k,\text{MIN}}$, $C_{k,\text{MAX}}$	Allowable concentration range of species k	$C_{\text{Glc}}, C_{\text{Lac}}, C_{\text{O2}} [=] \frac{\text{mol}}{\text{m}^3}$; $C_{\text{EPO}} [=] \frac{\text{U}}{\text{m}^3}$; $C_{\text{SCF}} [=] \frac{\text{mg}}{\text{m}^3}$	Misener et al. [24]
	$D_{k,i}$	Diffusivity of species k in region $i \in \{1, 2, 3\}$	m^2/s	Misener et al. [24]
	ε_{PU}; ε_{HF}	Porosity of HF and PU scaffold	$\varepsilon_{\text{PU}} = 0.79 \pm 0.1$; $\varepsilon_{\text{HF}} = 0.8$	Macedo [14]
	γ_h	Death rate (non-age) of differentiated cells; $h \in \{E, L, G\}$	$\gamma_E = 0.001$ days^{-1}; $\gamma_G = 0.15$ days^{-1}; $\gamma_L = 2.4$ days^{-1}	Colijn and Mackey [31]
	γ_Q	Death rate of HSC during proliferation	0.1 days^{-1}	Colijn and Mackey [31]
	H_{MAX}	Maximum cell density	0.5×10^{-6} cells / mm^3	Peng and Palsson [83]
	$\hat{J}_{\text{Cells}}$	Cellular flux across ceramic HF when $\Delta\hat{H}_{\text{Cells}} = 1.00 \times 10^6$ cells / mm^3	$\hat{J}_{\text{Cells}} = 5.76 \pm 2.25 \times 10^5$ cells / mm^2 / day	Macedo [14] p. 177
	p_k	Price of species k	$p_{\text{EPO}} = 0.023 \frac{\$}{\text{U}}$; $p_{\text{SCF}} = 320 \frac{\$}{\text{mg}}$	R&D Systems
	p_{UCB}	Price of umbilical cord blood	\$220 / 4.0×10^8 nucleated cells	NHS [84]
	$t_{1/2,k}$	Growth factor half-life	$t_{1/2,\text{EPO}} = 3$ days[1]; $t_{1/2,\text{SCF}} = 2$ days	Kishimoto et al. [85]
	τ_Q	Stem Cell Proliferation time	1.4 days	Colijn and Mackey [31]
	V_k	Maximum rxn rate of species k in the scaffold		Misener et al. [24]

continued on the next page

[1] An NHS pharmacist advised us that *the patient may . . . store [EPO] at room temperature for up to three days*

Table 3 (Definitions) continued

	Symbol	Description	Units	Source
			$V_{Glc}, V_{Lac}, V_{O2}\,[=]\,\frac{mol}{m^3\,day}$; $V_{EPO}\,[=]\,\frac{U}{m^3\,day}$; $V_{SCF}\,[=]\,\frac{mg}{m^3\,day}$	
Uncertain Parameters	$V_{O_2,c}$	Rxn rate of O_2 with cell type c in the scaffold		Chow et al.[67]
			$V_{O_2,HSC} = 3.89\times10^{-14}\,\frac{mol}{cell\,day}$; $V_{O_2,RBC} = 1.56\times10^{-13}\,\frac{mol}{cell\,day}$ $V_{O_2,Gran} = 1.56\times10^{-11}\,\frac{mol}{cell\,day}$; $V_{O_2,Leuk} = 1.2\times10^{-12}\,\frac{mol}{cell\,day}$	Colijn and Mackey[31]
			$V_{O_2,PSC} = V_{O_2,SC} = V_{O_2,MySC} = 3.89\times10^{-14}\,\frac{mol}{cell\,day}$ $V_{O_2,LySC} = 1.68\times10^{-14}\,\frac{mol}{cell\,day}$; $V_{O_2,CFU\text{-}GM} = V_{O_2,CFU\text{-}EO} = 1.56\times10^{-11}\,\frac{mol}{cell\,day}$ $V_{O_2,CFU\text{-}MEG} = 3.46\times10^{-11}\,\frac{mol}{cell\,day}$; $V_{O_2,BFU\text{-}E} = 1.56\times10^{-13}\,\frac{mol}{cell\,day}$	Lobato da Silva et al.[30]
Decision Variables	$C_{k,IN}$	Conc. of species k at BR inlet	$C_{Glc,IN}, C_{Lac,IN}, C_{O2,IN}\,[=]\,\frac{mol}{m^3}$; $C_{EPO,IN}\,[=]\,\frac{U}{m^3}$; $C_{SCF,IN}\,[=]\,\frac{mg}{m^3}$	Misener et al.[24]
	L	Length of BR	$\hat{L} = 150$ mm; $L \in [50$ mm, 200 mm$]$	Macedo[142]
	N_{HF}	Number of hollow fibers	$\hat{N}_{HF} = 8$; $N_{HF} \in \{1,\ldots,20\}$	Macedo[14]
	$N_{HF,CRM}$	Number of ceramic HF	$\hat{N}_{HF,CRM} = 4$; $N_{HF,CRM} \in \{1,\ldots,20\}$	Macedo[14]
	$N_{HF,PAN}$	Number of polymeric HF	$\hat{N}_{HF,PAN} = 4$; $N_{HF,PAN} \in \{1,\ldots,20\}$	Macedo[14]
	N_R	Number of equivalent BRs	$\hat{N}_R = 1$	Macedo[14]
	R_4	Radius of region $i = 4$ mm	$\hat{R}_4 = 3.75$ mm; $R_4 \in [2, 10]$	Macedo[14]
	U_Z	Bulk liquid velocity in the HF	$\hat{U}_Z = 7.1\times10^{-2}\,\frac{mm}{s}$; $U_Z \in [\hat{U}_Z, 2\cdot\hat{U}_Z]$	Macedo[14]
Dependent Variables	$\beta_{Q,w}$	Rate of entry into the proliferative phase	$days^{-1}$	Colijn and Mackey[31]
	$C_{k,i}(r, z)$	Conc. of species k in region i at location (r, z)	$C_{Glc}(r,z), C_{Lac}(r,z), C_{O2}(r,z)\,[=]\,\frac{mol}{m^3}$; $C_{EPO}(r,z)\,[=]\,\frac{U}{m^3}$; $C_{SCF}(r,z)\,[=]\,\frac{mg}{m^3}$	Misener et al.[24]
	$C_{k,OUT}$	Conc. of species k at BR outlet	$C_{Glc,OUT}, C_{Lac,OUT}, C_{O2,OUT}\,[=]\,\frac{mol}{m^3}$; $C_{EPO,OUT}\,[=]\,\frac{U}{m^3}$; $C_{SCF,OUT}\,[=]\,\frac{mg}{m^3}$	Misener et al.[24]

continued on the next page

[2]Variables marked with ˆ denote design choices of Macedo[14]

Table 3 (Definitions) continued

	Symbol	Description	Units	Source
	$\gamma_{P,w}$	Production rate of cells from the BR	days^{-1}	Misener et al.[24]
	k_h^e	Self-expansion rate constant	$k_h^e \in [0.0001$ / day, 1 / day$]$	Lobato da Silva et al.[30]
	k_h^d	Cell death rate constant	$k_h^d \in [0.0001$ / day, 1 / day$]$	Lobato da Silva et al.[30]
	k_h^k	Differentiation rate constant	$k_h^k \in [0.0001$ / day, 1 / day$]$	Lobato da Silva et al.[30]
	$H_{h,w}$	Cell types $h \in \{Q, E, L, G\}$ in region 3 for $w \in \{1, \ldots, 5\}$	cells / m^3	Misener et al.[24]
	$\kappa_{h,w}$	Differentiation rate towards committed lineages $h \in \{E, L, G\}$	days^{-1}	Colijn and Mackey[31]
Dependent Variables	R_3	Radius of region $i \in \{3\}$	mm	Misener et al.[24]
	r	Radial distance from centre of Krogh cylinder	mm	
	Rxn_k	Reaction Rate of Species k (0th-order approx) $\text{R}_{\text{Glc}}, \text{Rxn}_{\text{Lac}}, \text{Rxn}_{\text{O2}} [=] \frac{\text{mol}}{\text{m}^3\ \text{day}}$; $\text{Rxn}_{\text{EPO}} [=] \frac{\text{U}}{\text{m}^3\ \text{day}}$; $\text{Rxn}_{\text{SCF}} [=] \frac{\text{mg}}{\text{m}^3\ \text{day}}$		Misener et al.[24]
	τ_k	Rate for replenishing growth factor	–	Misener et al.[24]
	$\text{Vol}_{K,k}$	Krogh volume for each species $k \in \{\text{Glc, Lac, } O_2\text{, EPO, SCF}\}$	m^3	Misener et al.[24]
	Vol_R	Reactor volume	m^3	Misener et al.[24]
	Vol_T	Total volume	m^3	Misener et al.[24]
	z	Axial distance along the BR	mm	

Parameter Uncertainty and Sensitivity Analysis

Table 4: Range of uncertain parameters. Choices justified in Misener et al. [24]

Parameter	Units	Nominal Val	Range	Source
A_E	–	5.63×10^5	$\pm 50\%^\dagger$	Colijn and Mackey [31]
A_G	–	2.82×10^5	$\pm 50\%^\dagger$	Colijn and Mackey [31]
A_L	–	7.52×10^4	$\pm 50\%^\dagger$	Colijn and Mackey [31]
$\hat{C}_{\text{Glc,OUT}}$	mol / m^3	19.9	$\pm 50\%^\dagger$	Macedo, Yeo et al. [14,35]
$\hat{C}_{\text{Lac,OUT}}$	mol / m^3	13.5	$\pm 50\%^\dagger$	Macedo, Yeo et al. [14,35]
$D_{\text{Glc},3}$	m^2 / s	8.39×10^{-10}	$\pm 0.52 \times 10^{-10}$	Maxwell [86]
$D_{\text{Lac},3}$	m^2 / s	9.01×10^{-10}	$\pm 0.54 \times 10^{-10}$	Maxwell [86]
$D_{\text{O2},3}$	m^2 / s	2.98×10^{-9}	$\pm 0.13 \times 10^{-9}$	Maxwell [86]
$D_{\text{EPO},3}$	m^2 / s	0.99×10^{-10}	$\pm 0.25 \times 10^{-10}$	Maxwell [86]
$D_{\text{SCF},3}$	m^2 / s	1.21×10^{-10}	$\pm 0.30 \times 10^{-10}$	Maxwell [86]
ε_{HF}	–	0.8	$\pm 50\%^\dagger$	Macedo [14]
γ_E	days^{-1}	0.001	$\pm 50\%^\dagger$	Colijn and Mackey [31]
γ_G	days^{-1}	0.15	$\pm 50\%^\dagger$	Colijn and Mackey [31]
γ_L	days^{-1}	2.4	$\pm 50\%^\dagger$	Colijn and Mackey [31]
γ_Q	days^{-1}	0.1	$\pm 50\%^\dagger$	Colijn and Mackey [31]
$\hat{J}_{\text{Cells}}$	cells / mm^2 / day	5.76	± 2.25	[14] p. 177
$\kappa_{E,w}^{\text{LO}}$	1 / day	0.023	$\pm 50\%^\dagger$	Colijn and Mackey [31]
$\kappa_{E,w}^{\text{UP}}$	1 / day	1	$\pm 50\%^\dagger$	Colijn and Mackey [31]
$\kappa_{G,w}^{\text{LO}}$	1 / day	0.023	$\pm 50\%^\dagger$	Colijn and Mackey [31]
$\kappa_{L,w}^{\text{LO}}$	1 / day	0.023	$\pm 50\%^\dagger$	Colijn and Mackey [31]
p_{EPO}	\$ / U	0.023	$\pm 50\%^\dagger$	Timmins and Nielsen [70]
p_{SCF}	\$ / mg	320	$\pm 50\%^\dagger$	Timmins and Nielsen [70]
p_{UCB}	\$ / 4.0×10^8 nucleated cells	220	$\pm 50\%^\dagger$	NHS [84]
$t_{1/2,\text{EPO}}$	days	3	$\pm 50\%^\dagger$	NHS[3]
$t_{1/2,\text{SCF}}$	days	2	$\pm 50\%^\dagger$	Kishimoto et al. [85]
τ_Q	days	1.4	$\pm 50\%^\dagger$	Colijn and Mackey [31]
		Range		Chow et al. [67]
$V_{\text{O}_2,\text{HSC}}$	mol · cell / day	$3.89 - 15.58 \times 10^{-14}$		
$V_{\text{O}_2,\text{RBC}}$	mol · cell / day	$3.89 - 15.58 \times 10^{-14}$		
$V_{\text{O}_2,\text{Gran}}$	mol · cell / day	$52.8 - 1557.6 \times 10^{-14}$		
$V_{\text{O}_2,\text{Leuk}}$	mol · cell / day	$1.68 - 120.0 \times 10^{-14}$		

† Parameters without published uncertainty ranges vary ±50%

[3] An NHS pharmacist advised us that *the patient may . . . store [EPO] at room temperature for up to three days*

Table 5: Of the 30 parameters, 9 induced the global optimum to vary by 10% or more with respect to S_i defined in Eq. (3)

Uncertain Parameter	Description	Sensitivity
$\hat{J}_{\text{Cells}}$	Cellular flux across ceramic HF	36.34
$t_{1/2,\text{SCF}}$	SCF growth factor half-life	32.01
p_{SCF}	Price of species SCF	25.06
A_E	Amplification parameter for RBC differentiation	20.03
$V_{\text{O}_2,\text{Gran}}$	Rxn rate of O_2 with cell type Gran in the scaffold	18.82
$t_{1/2,\text{EPO}}$	EPO growth factor half-life	18.59
ε_{HF}	Porosity of hollow fiber	16.81
p_{EPO}	Price of species EPO	14.52
V_{Glc}	Maximum rxn rate of species Glc in the scaffold	13.16

This section considers the 30 uncertain parameters in Table 4; we define the single- and multi-variate sensitivity analysis. Parameters with known error bars vary within their expected uncertainty levels; the remaining parameters were allowed to take values 50% (L1), 90% (L2), 110% (U1), and 150% (U2) of their nominal values. Define the optimal point of the optimization algorithm to be $f^*(\mathbf{x}, \mathbf{u})$ where variables $\mathbf{x}$ are the design decisions and $\mathbf{u} \subset \mathbb{R}^p$ are uncertain parameters. Eq. (2) introduces $\Delta_i^{f^*}(\mathbf{x}, \mathbf{u}, \delta_{i,\ell})$ as a quantity similar to an elementary effect and normalizes it as the percent difference from the nominal optimal value; $\delta_{i,\ell}$ perturbs uncertain parameter from its nominal u_i value with respect to levels $\ell \in \{\text{L1}, \text{L2}, \text{U1}, \text{U2}\}$. We also define the sensitivity index S_i in Eq. (3):

$$\Delta_i^{f^*}(\mathbf{x}, \mathbf{u}, \delta_{i,\ell}) = \frac{100}{f^*(\mathbf{x}, \mathbf{u})} \cdot \left[f^*(\mathbf{x}, u_1, \ldots, u_i + \delta_{i,\ell}, \ldots, u_p) - f^*(\mathbf{x}, \mathbf{u}) \right] \qquad \forall i \qquad (2)$$

$$S_i = \frac{1}{4} \sum_{\ell \in \{\text{L1},\text{L2},\text{U1},\text{U2}\}} \Delta_i^{f^*}(\mathbf{x}, \mathbf{u}, \delta_{i,\ell}) \qquad \forall i \qquad (3)$$

Table 5 lists the uncertain parameters with sensitivity indices $S_i > 10\%$. We also define 2D parameter variation $\Delta_{i,j}^{f^*}(\mathbf{x}, \mathbf{u}, \delta_{i,\ell}, \delta_{j,\ell 2})$ in Eq. (4) and sensitivity in Eq. (5).

$$\Delta_{i,j}^{f^*}(\mathbf{x}, \mathbf{u}, \delta_{i,\ell 1}, \delta_{j,\ell 2}) = \frac{100}{f^*(\mathbf{x}, \mathbf{u})} \cdot [f^*(\mathbf{x}, u_1, \ldots, u_i + \delta_{i,\ell 1}, \ldots, u_j + \delta_{j,\ell 2}, \ldots, u_p) - \qquad (4)$$

$$f^*(\mathbf{x}, \mathbf{u})] \qquad \forall i \neq j$$

Table 6: 12 combinations inducing $>15\%$ change beyond what we expect from the linear analysis; sensitivity $S_{i,j}$ is defined in Eq. (5). Note that 13 of the 24 entries contributing to inducing $>15\%$ change are related to cellular kinetics (**blue bold**) and an additional 7 parameter entries are related to cellular consumption of nutrients and production of waste.

Parameter 1	Description	Parameter 2	Description	Sensitivity
$\boldsymbol{\kappa_{E,w}^{\mathbf{LO}}}$	RBC differentiation	$\boldsymbol{\kappa_{L,w}^{\mathbf{LO}}}$	Lymphocyte differentiation	39.5%
$\mathbf{A_E}$	RBC amplification	$\boldsymbol{\kappa_{L,w}^{\mathbf{LO}}}$	Lymphocyte differentiation	27.8%
$\mathbf{A_E}$	RBC amplification	$\boldsymbol{\kappa_{E,w}^{\mathbf{UP}}}$	RBC differentiation	19.8%
$t_{1/2,\mathrm{SCF}}$	SCF half-life	$\varepsilon_{\mathrm{HF}}$	HF porosity	19.0%
$V_{\mathrm{O_2,Gran}}$	O_2 rxn with Gran	$\mathbf{A_E}$	RBC amplification	18.8%
$V_{\mathrm{O_2,Gran}}$	O_2 rxn with Gran	$\hat{C}_{\mathrm{Lac,Out}}$	Lactate output	17.9%
$V_{\mathrm{O_2,RBC}}$	O_2 rxn with RBC	$\mathbf{A_E}$	RBC amplification	17.0%
$\mathbf{A_E}$	RBC amplification	$\mathbf{A_G}$	Granulocyte amplification	16.4%
$V_{\mathrm{O_2,RBC}}$	O_2 rxn with RBC	$\hat{C}_{\mathrm{Lac,Out}}$	Lactate output	15.7%
$t_{1/2,\mathrm{SCF}}$	SCF half-life	$J_{\mathrm{Cells},w}$	Cellular flux	15.7%
$V_{\mathrm{O_2,RBC}}$	O_2 rxn with RBC	$\boldsymbol{\kappa_{L,w}^{\mathbf{LO}}}$	Lymphocyte differentiation	15.7%
$\mathbf{A_E}$	RBC amplification	$\mathbf{A_L}$	Lymphocyte amplification	15.3%

$$S_{i,j} = \frac{1}{16} \sum_{\ell 1,\ell 2 \in \{\mathrm{L1,L2,U1,U2}\}} |\Delta_{i,j}^{f^*}(\mathbf{x},\,\mathbf{u},\,\delta_{i,\ell 1},\,\delta_{j,\ell 2}) - \Delta_i^{f^*}(\mathbf{x},\,\mathbf{u},\,\delta_{i,\ell 1}) - \Delta_j^{f^*}(\mathbf{x},\,\mathbf{u},\,\delta_{j,\ell 2})| \ \forall\, i \neq j \tag{5}$$

Calculating $S_{i,j}$, we find that 41 combinations of 2 variables induce $>10\%$ change beyond what we expect from the linear analysis and 12 combinations inducing $>15\%$ change. For a linear optimization model we would expect the sensitivity $S_{i,j}$ to be approximately 0; significantly large values of $S_{i,j}$ indicate that nonlinearity in the optimization model is affecting the final outcome.

Robust Optimization Counterpart

This section first develops a robust counterpart model for oxygen consumption in the BR; we explain the implications in some level of detail. We also briefly develop the remaining robust counterparts for the uncertain parameters in Table 4; Figure 7 diagrams the ideas.

Oxygen Consumption: Oxygen consumption V_{O_2} is the sum of $V_{O_2,h}$ for each type of hematopoietic cell h weighted by cellular concentration H_h:

$$V_{O_2} = \sum_h V_{O_2,h} \cdot H_h \tag{6}$$

Robust optimization only applies to inequalities, so the equality in Eq. (6) becomes $\geq$; the BR is O_2 limited and therefore a global optimization algorithm will drive the total oxygen consumption V_{O_2} as low as possible and just including the $\geq$ side is effectively the same as the equality because any BR not consuming the maximum amount of oxygen available would automatically improve itself by consuming more oxygen. We subsequently define the (ε, δ)-Interval Robust Counterpart[65]:

$$\sum_h V_{O_2,h} \cdot H_h + \varepsilon \cdot \sum_h |V_{O_2,h}| \cdot |H_h| - V_{O_2} \leq \delta$$

where $\varepsilon \geq 0$ is the relative uncertainty level, $|\hat{V}_{O_2,h} - V_{O_2,h}| = \varepsilon \; |V_{O_2,h}|$, defining the range between nominal parameter values, $V_{O_2,h}$, and worst-case parameter realizations, $\hat{V}_{O_2,h}$, and $\delta \geq 0$ is the infeasibility tolerance. Note here that $V_{O_2,h}, H_{h,w} > 0 \; \forall \; h$, so we can simplify to:

$$(1+\varepsilon) \cdot \sum_h V_{O_2,h} \cdot H_h - V_{O_2} \leq \delta \tag{7}$$

Setting $(\varepsilon = 0, \delta = 0)$ in Eq. (7) corresponds to the nominal optimization model which does not incorporate uncertainty; a worst-case error analysis corresponds to setting ε to its maximum value and $\delta = 0$. There are robust optimization methods which select levels of ε and δ to achieve a desired trade-off between a nominal and worst-case model, but we do not have enough data for the RBC BR to accurately parameterize ε and δ; we therefore consider the nominal and worst-case models separately and in comparison to each other.

Glucose Consumption $V_{\mathbf{Glc},w}$ / Lactate Production $V_{\mathbf{Lac},w}$: Original equation in model formulation is based on experimental data:

$$V_{k,w} = \frac{\hat{U}_Z \cdot \left(\hat{C}_{k,\mathrm{IN}} - \hat{C}_{k,\mathrm{OUT}}\right)}{\hat{L}} \cdot \frac{\sum_h H_{h,w}}{H_{\mathrm{MAX}}} \quad \forall \; k \in \{\mathrm{Glc}, \mathrm{Lac}\}$$

To transform into the robust counterpart, we begin by observing that the equalities can equivalently be the inequalities that make the equation as bad as possible:

$$V_{\mathrm{Glc},w} \geq \frac{\hat{U}_Z \cdot \left(\hat{C}_{\mathrm{Glc,IN}} - \hat{C}_{\mathrm{Glc,OUT}}\right)}{\hat{L}} \cdot \frac{\sum_h H_{h,w}}{H_{\mathrm{MAX}}}$$
$$V_{\mathrm{Lac},w} \leq \frac{\hat{U}_Z \cdot \left(\hat{C}_{\mathrm{Lac,IN}} - \hat{C}_{\mathrm{Lac,OUT}}\right)}{\hat{L}} \cdot \frac{\sum_h H_{h,w}}{H_{\mathrm{MAX}}}$$

Rearranging:

$$\frac{\hat{U}_Z \cdot \left(\hat{C}_{\text{Glc,IN}} - \hat{C}_{\text{Glc,OUT}}\right)}{\hat{L}} \cdot \frac{\sum_h H_{h,w}}{H_{\text{MAX}}} - V_{\text{Glc},w} \leq 0$$
$$-\frac{\hat{U}_Z \cdot \left(\hat{C}_{\text{Lac,IN}} - \hat{C}_{\text{Lac,OUT}}\right)}{\hat{L}} \cdot \frac{\sum_h H_{h,w}}{H_{\text{MAX}}} + V_{\text{Lac},w} \leq 0$$

Adding the uncertainty:

$$\frac{\hat{U}_Z}{\hat{L}} \cdot \left[\ \hat{C}_{\text{Glc,IN}} - \hat{C}_{\text{Glc,OUT}} + \hat{C}_{\text{Glc,UN}}\right] \cdot \frac{\sum_h H_{h,w}}{H_{\text{MAX}}} - V_{\text{Glc},w} \leq \delta$$
$$\frac{\hat{U}_Z}{\hat{L}} \cdot \left[-\hat{C}_{\text{Lac,IN}} + \hat{C}_{\text{Lac,OUT}} + \hat{C}_{\text{Lac,UN}}\right] \cdot \frac{\sum_h H_{h,w}}{H_{\text{MAX}}} + V_{\text{Lac},w} \leq \delta$$

Mass Transfer: None of the species mass transfer parameters (diffusion; porosity) change the baseline noticeably (by more than $\approx 10\%$) when varying parameters, so we will not consider robustifying the design with respect to these parameters.

Cell Flux Across Membrane: This parameter strongly affects final price, so we take the original equation:

$$\gamma_{P,w} = \frac{\hat{J}_{\text{Cells}}}{\Delta\hat{H}_{\text{Cells}}} \cdot \frac{\pi \cdot N_{\text{HF,CRM}} \cdot R_{2,\text{CRM}} \cdot L}{\text{Vol}_R}\ \forall\, w \in \{1, \ldots, 5\}$$

and replace it with an equivalent inequality:

$$\gamma_{P,w} \leq \frac{\hat{J}_{\text{Cells}}}{\Delta\hat{H}_{\text{Cells}}} \cdot \frac{\pi \cdot N_{\text{HF,CRM}} \cdot R_{2,\text{CRM}} \cdot L}{\text{Vol}_R}\ \forall\, w \in \{1, \ldots, 5\}$$

This works because the optimization model is going to want to make $\gamma_{P,w}$ as large as possible. Rearranging:

$$\gamma_{P,w} - \frac{\hat{J}_{\text{Cells}} - J^{\text{U}}_{\text{Cells}}}{\Delta\hat{H}_{\text{Cells}}} \cdot \frac{\pi \cdot N_{\text{HF,CRM}} \cdot R_{2,\text{CRM}} \cdot L}{\text{Vol}_R} \leq \delta\ \forall\, w \in \{1, \ldots, 5\}$$
